# Supplementary material for: What are the perspectives of adults aged 18–40 living with type 2 diabetes in urban settings towards barriers and opportunities for better health and well-being: a mixed-methods study
Source: BMJ Open. 2023 Sep 20;13(9):e068765. doi: 10.1136/bmjopen-2022-068765 (PMC10514606; doi:10.1136/bmjopen-2022-068765)
Supplement: Supplementary data [file bmjopen-2022-068765supp001.pdf]

Appendix 1. Q-sort statements Greater Manchester factor array

	Factor 1	Factor 2	Factor 3	Factor 4	Factor 5
1 Diabetes is the least of my worries	-4	-4	0	-6	2
2 I worry about the cost of my diabetes	-3	3	-4	0	0
3 The way I make decisions about my healthcare is mostly dependent on how much money I have	-1	4	-2	3	-3
4 When it comes to food, I usually sacrifice quality for quantity	-1	-2	-4	5	6
5 Healthy foods are a luxury	-1	3	-1	1	1
6 Spending money on exercise classes or the gym is something I find difficult to do	1	5	-2	5	-1
7 Not having enough time makes people sick	1	2	-1	-4	-1
8 If I had more time, I'd exercise regularly	0	1	0	0	4
9 I just don't have the time to buy and cook healthy food	-4	1	-4	0	0
10 Diabetes support groups may be helpful, but I don't have time to participate	1	-1	2	6	-1
11 Taking care of others takes up most of my free time	0	5	-2	-2	2
12 I just don't have the skills to cook healthy meals	-3	-2	-5	1	3
13 For me, it's easy to learn about diabetes	4	0	3	-1	4
14 I think that diabetes or health-related support groups are pointless	-4	-3	0	-5	0
15 Where I live, fresh groceries are hard to get	-5	-3	-4	0	5
16 Living in the city is stressful and harmful to my health and wellbeing	-4	-1	1	-1	-5
17 I'd rather live elsewhere, but I have to live where I do because of work	-3	-4	4	2	-4
18 I am quite concerned about air pollution	0	0	0	3	-3
19 I think that where I live, it's not very safe to be out alone	-1	3	-5	-3	3
20 The climate where I live makes it really hard for me to want to walk or go somewhere by bicycle	-1	-1	-3	-1	-5
21 For me, it is important that I can get around by bike or on foot easily	0	0	-1	1	0
22 I like certain foods or dishes especially because they remind me of my childhood	1	1	1	4	-3
23 Cooking for those I love is a way of showing that I care for them	0	0	5	3	-1
24 When my family or my friends and I meet, we eat!	3	1	2	-1	-3
25 When I am by myself, I often eat food that is not very healthy	3	6	1	1	1
26 When I eat out together with friends, I usually end up eating unhealthy foods	6	4	3	-4	3
27 I often feel lonely	-5	2	2	6	2
28 To me, food is about more than just calorie intake	2	4	6	-1	3
29 Society puts a lot of pressure on women to be slim	5	5	-2	4	-3
30 I need my friends and social life to feel happy	4	0	-1	-2	-4
31 Being alone does not mean being lonely	4	2	3	0	-2
32 I tend to prioritise the needs of others over my own	3	6	0	1	-4
33 Men tend to be less interested in taking care of themselves than women	-2	-1	2	1	1
34 I have some health concerns that are more important to me than my diabetes	-2	0	1	-3	2
35 With today's medicine, diabetes is not a scary illness anymore	2	-2	3	-6	0
36 Diabetes is a disease of overweight people	-2	-5	2	-2	0
37 It is my fault that I now have diabetes	1	1	6	0	5
38 If I had known what having diabetes is like I would have tried harder to avoid becoming diabetic	2	3	0	3	-1
39 If I really had to, I could adjust to a healthy lifestyle	5	2	4	4	-2
40 I usually do what my doctor tells me to do	6	0	-1	-5	4
41 I avoid socialising because of my diabetes	-6	-3	-6	2	-1
42 I find it helpful to talk to other people about my diabetes	3	-2	-3	4	1
43 The modern world makes us ill	-1	1	3	1	-2
44 Feeling integrated with a community is important to me	1	-3	-1	3	1
45 Changes in my neighbourhood worry me	0	-1	0	0	4
46 I don't really trust health services or doctors	-6	-2	0	-5	0
47 Everybody just seems to be a bit bigger nowadays	1	0	5	0	2
48 My weight does not matter to me because I feel like a healthy person	-2	-6	-2	-3	5
49 Overweight people are often lazy	-2	-3	5	2	0
50 I am too embarrassed to go to the gym because of how I look	-1	2	0	-1	-5
51 Compared to others, I take good care of myself	0	-4	-3	-4	-2
52 I know who I can trust with my healthcare	2	0	-1	-2	1
53 I generally trust the government	0	-5	-6	-3	6
54 'Fitness freaks' have no joy in life	-3	-4	-3	2	2
55 I think that diabetes is inherited	2	1	1	-1	0
56 I think that diabetes is a death sentence	-5	-2	-2	2	-6
57 Most days, I feel good about my future	3	-1	-3	-2	3
58 At this point in my life, I feel like the choices I make are my own	5	4	4	-3	1
59 I get annoyed or upset with myself if I don't do enough for my health	2	3	1	5	-2
60 If I decide to be overweight or not exercise enough, that should be my choice	0	2	2	0	0
61 We owe it to society to be fit and healthy	0	0	1	-3	-4
62 Being unhealthy means you've given up on life	-3	-5	0	2	-1
63 Managing diabetes well is simply a matter of making the right choices	4	-1	4	-2	-2
64 My church is my rock	-2	-6	-5	0	-6
**NB: Distinguishing units are coloured**					
