# Supplementary material for: What are the perspectives of adults aged 18–40 living with type 2 diabetes in urban settings towards barriers and opportunities for better health and well-being: a mixed-methods study
Source: BMJ Open. 2023 Sep 20;13(9):e068765. doi: 10.1136/bmjopen-2022-068765 (PMC10514606; doi:10.1136/bmjopen-2022-068765)
Supplement: Supplementary data [file bmjopen-2022-068765supp002.pdf]

Appendix 2. Interviews and focus groups topic guide

Session	Areas to Be Covered	Example Questions/ Prompts<br>(Not to be considered in any way exhaustive. Should be reformatted for ease of use in Focus Group or Interview session and language adapted if needed)
Introductory Session		
Open discussion around Q-Sort, content of statements, insights gained through procedure	Content of statements and nature of Q-sort	Principally unstructured discussion to give them an opportunity to voice what matters to them (and to allow participants to familiarise themselves with others in the Focus Groups).<br><br>If needed, specific sample statements may be presented as well as any qualitative comments already made. If needed, questions or prompts might include:<br>• How did you feel about X statement – is it easy to agree with/ disagree with? Why?<br>• Were there any statements that really spoke to you/ that really made you annoyed or sad? Why?<br>• Were there any statements that you did not understand or that you thought were just a bit silly? Why?<br>• Can you think of any statements that you thought were missing?<br>• The statements were based on interviews that we conducted with people in [CCD cities]. In what way do you think might their experience be similar or different to yours? Example?<br>• Were there any statements that made you reflect on how you yourself /your living with diabetes? Which ones?<br>• Did the sorting process make you think/ feel differently about yourself and your diabetes – if yes, why?<br>• Are there things we could have done differently with the Q-sort?
Discussion: Q-Sort findings		
Semi-structured discussion of findings with participants		This session provides an opportunity to deepen findings and to gather data for factor description and interpretation, as well as to identify any areas of relevance not covered by the statements. It should also be used to explore qualitatively areas of specific interest or priority. Finally, it is generally good practice to allow participants to comment on findings before finalising results.
	'Validity' of factors and exemplars	• How do you feel about [insert *defining* statements of groups]? Why?<br>• When you think about the groups (factors) that we have identified, do they seem typical/ plausible/ relevant/ well described? Why?<br>• How would you describe the various viewpoints associated with each group? Can you describe a person when you hear about these viewpoints? What would that person be like?

Appendix 2. Interviews and focus groups topic guide

		• What do you think of the names that we have given the groups?<br>• What do you think of the exemplars that we have described – do our descriptions make sense, do you think that this is informative/ accurate?<br>• Based on what we have presented to you, what would you say most characterizes/ differentiates the groups regarding their experience of diabetes? How does that relate to your own experiences?
	Relevance	• In what way do you think do the groups that we identified have different priorities/ abilities/ needs regarding health, wellbeing, and living with diabetes?<br>• In what way might our research findings (the groups and exemplars, description, interpretation) be useful when trying to improve diabetes care/ prevention?
	Barriers/ promoters	• How would you say do the different groups face different challenges regarding living with diabetes? What are those challenges; do you share them?<br>• What could be improved in terms of reducing [main stated barriers]?
	Social factors cultural determinants	Additional suggestion: provide list and description of social factors and cultural determinants and encourage discussion around which apply to what group; what their relevance and impact may be on each group, and how to ameliorate potentially negative impact.
Discussion: Diabetes in My City<br>Semi-structured discussion around lived experiences of participants in Greater Manchester as relevant to health, wellbeing, and diabetes		• Would you say that Greater Manchester is a good place to live for someone with diabetes? Why?<br>• Our physical activity level is important when it comes to living with diabetes. In what way does Greater Manchester make it easy or hard to be physically active? DO you have suggestions on how to change that?<br>• (If applicable: we have already discussed some things that may make living with diabetes in Greater Manchester difficult. Can you provide some more examples? How may we concretely address such issues?)<br>• If you could tell someone from [the city government/ local healthcare provider/ insurance (i.e. any potential stakeholders who may attend later session)] about something concrete they could do to improve your experience of living with diabetes, what would it be?
