# Supplementary material for: What are the perspectives of adults aged 18–40 living with type 2 diabetes in urban settings towards barriers and opportunities for better health and well-being: a mixed-methods study
Source: BMJ Open. 2023 Sep 20;13(9):e068765. doi: 10.1136/bmjopen-2022-068765 (PMC10514606; doi:10.1136/bmjopen-2022-068765)
Supplement: Supplementary data [file bmjopen-2022-068765supp003.pdf]

Appendix 3. Example RAP sheet

**Factor 1**	
**FGD/I participant(s):**<br>ID#<br>ID#	
**Q-sort comment(s):**<br>ID#<br>ID#<br>ID#<br>ID#	
**Analysis by:**	
**Diabetes journey**	
**Management**	
**GM lived experience**	
**Recommendations**	
**Notes (e.g. this interview a good source about x issue/standout quotations)**<br><br>ID#: “quote from transcript”<br><br>ID#: “quote from transcript”	
