# Supplementary material for: What are the perspectives of adults aged 18–40 living with type 2 diabetes in urban settings towards barriers and opportunities for better health and well-being: a mixed-methods study
Source: BMJ Open. 2023 Sep 20;13(9):e068765. doi: 10.1136/bmjopen-2022-068765 (PMC10514606; doi:10.1136/bmjopen-2022-068765)
Supplement: Author's manuscript [file bmjopen-2022-068765.draft_revisions.pdf]

BMJ Open

BMJ Open is committed to open peer review. As part of this commitment we make the peer review history of every article we publish publicly available.

When an article is published we post the peer reviewers' comments and the authors' responses online. We also post the versions of the paper that were used during peer review. These are the versions that the peer review comments apply to.

The versions of the paper that follow are the versions that were submitted during the peer review process. They are not the versions of record or the final published versions. They should not be cited or distributed as the published version of this manuscript.

BMJ Open is an open access journal and the full, final, typeset and author-corrected version of record of the manuscript is available on our site with no access controls, subscription charges or pay-per-view fees (http://bmjopen.bmj.com).

If you have any questions on BMJ Open's open peer review process please email info.bmjopen@bmj.com

BMJ Open

Understanding the health impact of social and cultural influences on people aged 18-40 living with type 2 diabetes: a mixed-methods study

Journal:	*BMJ Open*
Manuscript ID	bmjopen-2022-068765
Article Type:	Original research
Date Submitted by the Author:	04-Oct-2022

Complete List of Authors:	Croke, Sarah; The University of Manchester,<br>Volkmann, Anna-Maria; University College London, Anthropology<br>Perry, Catherine ; The University of Manchester, Division of Population Health, Health Services Research and Primary Care<br>Atkinson, Ross; University of Manchester, Manchester, M13 9PL , Division of Nursing, Midwifery and Social Work, School of Health Sciences, Faculty of Biology, Medicine and Health;<br>Pruneddu, Alessio; Independent research consultant<br>Morris, Lydia; The University of Manchester, Division of Psychology and Mental Health, School of Health Sciences<br>Bower, Peter; University of Manchester, NPCRDC

Keywords:	PRIMARY CARE, QUALITATIVE RESEARCH, DIABETES & ENDOCRINOLOGY

SCHOLARONE™ Manuscripts

I, the Submitting Author has the right to grant and does grant on behalf of all authors of the Work (as defined in the below author licence), an exclusive licence and/or a non-exclusive licence for contributions from authors who are: i) UK Crown employees; ii) where BMJ has agreed a CC-BY licence shall apply, and/or iii) in accordance with the terms applicable for US Federal Government officers or employees acting as part of their official duties; on a worldwide, perpetual, irrevocable, royalty-free basis to BMJ Publishing Group Ltd ("BMJ") its licensees and where the relevant Journal is co-owned by BMJ to the co-owners of the Journal, to publish the Work in this journal and any other BMJ products and to exploit all rights, as set out in our licence.

The Submitting Author accepts and understands that any supply made under these terms is made by BMJ to the Submitting Author unless you are acting as an employee on behalf of your employer or a postgraduate student of an affiliated institution which is paying any applicable article publishing charge ("APC") for Open Access articles. Where the Submitting Author wishes to make the Work available on an Open Access basis (and intends to pay the relevant APC), the terms of reuse of such Open Access shall be governed by a Creative Commons licence – details of these licences and which Creative Commons licence will apply to this Work are set out in our licence referred to above.

Other than as permitted in any relevant BMJ Author's Self Archiving Policies, I confirm this Work has not been accepted for publication elsewhere, is not being considered for publication elsewhere and does not duplicate material already published. I confirm all authors consent to publication of this Work and authorise the granting of this licence.

For peer review only

- Title of the article

"Understanding the health impact of social and cultural influences on people aged 18-40 living with type 2 diabetes: a mixed-methods study"

- Full name, postal address and e-mail of the corresponding author

1. Dr Sarah Croke, PhD

Centre for Primary Care and Health Services Research, Division of Population Health, Health Services Research & Primary Care, School of Health Sciences, Faculty of Biology, Medicine and Health, University of Manchester, Suite 1 6th Floor Williamson Building, Oxford Road, Manchester, UK, M13 9PL. tel: 07932 011581 email: sarah.croke@manchester.ac.uk

- Full name, department, institution, city and country of all co-authors

2. Dr Anna-Maria Volkmann, PhD

Department of Anthropology, University College London, London, UK. email: a.volkmann@ucl.ac.uk

3. Dr Catherine Perry, PhD

NIHR Applied Research Collaboration Greater Manchester (NIHR ARC GM), Division of Population Health, Health Services Research & Primary Care/Division of Nursing, Midwifery & Social Work, School of Health Sciences, Faculty of Biology, Medicine and Health, University of Manchester, Manchester, UK. email: catherine.perry@manchester.ac.uk

4. Dr Ross A. Atkinson, PhD

NIHR Applied Research Collaboration Greater Manchester (NIHR ARC GM), Division of Nursing, Midwifery & Social Work, School of Health Sciences, Faculty of Biology, Medicine and Health, University of Manchester, Manchester, UK. email: ross.atkinson@manchester.ac.uk

5. Dr Alessio Pruneddu, PhD

Independent research consultant, Salford, UK. email: info@qsortouch.com

6. Dr Lydia Morris, PhD

Division of Psychology and Mental Health, School of Health Sciences, Faculty of Biology, Medicine and Health, University of Manchester, Manchester, UK. email: lydia.morris@manchester.ac.uk

7. Professor Peter Bower, PhD

Centre for Primary Care and Health Services Research, Division of Population Health, Health Services Research & Primary Care, School of Health Sciences, Faculty of Biology, Medicine and Health, University of Manchester, Manchester, UK. email: peter.bower@manchester.ac.uk

- Word count, excluding title page, abstract, references, figures and tables

4304 (includes participant quotes as results).

For peer review only - http://bmjopen.bmj.com/site/about/guidelines.xhtml

CCD/SC/Manuscript_Final_(1)

"Understanding the health impact of social and cultural influences on people aged 18-40 living with type 2 diabetes: a mixed-methods study"

Sarah Croke, Anna-Maria Volkmann, Catherine Perry, Ross A. Atkinson, Alessio Pruneddu, Lydia Morris, and Peter Bower.

Abstract

Objectives: Delivered as part of the global assessment of diabetes in urban settings, this study explores different aspects of living in Greater Manchester for adults aged 18-40 with type 2 diabetes. Primary questions were: 1) can we identify sub-groups of adults under 40 years old sharing specific perspectives towards health, wellbeing and living with type 2 diabetes; and 2) do these perspectives reveal specific barriers to and opportunities for better type 2 diabetes prevention and management and improved wellbeing?

Design*:* The study employed a mixed method design with data collected through demographic questionnaires, Q-sort statement sorting exercises, focus groups discussions and individual interviews.

Participants*:* Those aged between 18 and 40, with a confirmed type 2 diabetes diagnosis, and living in Greater Manchester were eligible to participate. A total of 46 people completed the Q-sort exercise and 43 were included in the final analysis. Of those, 29 (67%) identified as female and 32 (75%) as white. Most common time since diagnosis was between five and ten years.

Results: The Q-sort analysis categorised 35 of the 43 participants (81%) into five sub-groups. Based on average statement sorts for each sub-group, perspectives were characterised as: 1) Stressed and calamity coping (n=13), 2) Financially disadvantaged and poorly supported (n=12), 3) Well-intentioned but not succeeding (n=5), 4) Withdrawn and worried (n=2), 5) Young and stigmatised (n=3). Holistic analysis of our qualitative data also identified some common issues across these sub-groups.

Conclusions: Adults under 40 with type 2 diabetes are not a homogenous group, but fall into five identifiable sub-groups. They also experience issues specific to this age group that make it particularly difficult for them to focus on their own health. More tailored support could help them to make the necessary lifestyle changes and manage their type 2 diabetes better.

For peer review only - http://bmjopen.bmj.com/site/about/guidelines.xhtml

Article Summary

Strengths and limitations of this study

- A key feature of a Q-study is the ability to reveal shared perspectives among a group of participants, and for those to be understood holistically.
- The Q-sort requires a minimum of 35 participants to ensure statistical validity and a maximum of 64 (i.e. not exceeding the number of statements).
- The challenge to recruit younger adults living with type 2 diabetes as participants for this study were addressed by utilising multiple routes, including social media.
- Restrictions due to the COVID-19 pandemic forced us to conduct all data collection via online platforms; this limited some from participating while providing opportunity to others.
- Zabala's 'qmethod' analysis package allowed us to extract factors and thereby establish shared perspectives in our sample that met statistical significance, despite sometimes having smaller numbers within some sub-groups.

For peer review only

Introduction

Type 2 diabetes is one of the most common metabolic disorders worldwide [1] and leads to complications that cause both psychological and physical distress and put a huge burden on healthcare systems [2]. One response to this, in recent years, has been the Cities Changing Diabetes programme, a programme advocating for urgent action against type 2 diabetes on a global scale. Launched in 2015, via a partnership between Novo Nordisk, University College London and Steno Diabetes Center, and implemented by City Partners, it aims to map the challenge, share solutions and act on the growing impact of type 2 diabetes in some of the world's great cities [3]. In 2019, Greater Manchester, UK became a City Partner.

A report conducted as part of this programme using the 'Rule of Halves' approach [4, 5] identified patterns of diagnosis and treatment in Greater Manchester and indicated that type 2 diabetes in the area is under-diagnosed in adults under 40 years of age. This also showed that, where type 2 diabetes was diagnosed, those under 40 seem less likely to receive appropriate care and achieve HbA1c treatment targets when compared with older age groups.

Previous research suggests that adults aged 18-40 with type 2 diabetes have higher physical morbidity and mortality than other type 2 diabetes sub-groups and poorer outcomes than their older counterparts [6]. People in this group are also more likely to be obese with sub-optimal glycaemic control and have a high risk of developing at least one severe diabetes complication by the time they are in their 40s, reducing life expectancy by 15 years [6]. A further concern is poor adherence to medication [7, 8]. Being a younger age at diagnosis is also associated with higher risk of mortality and vascular disease [9], while people may require more intensive psychological and self-care support compared with those who develop type 2 diabetes later [6]. It is suggested that targeting younger patients, earlier in their trajectory, can present a unique opportunity to improve longer term outcomes [10].

We aimed to explore the following questions for adults under 40 with type 2 diabetes, living in Greater Manchester:

- Can we identify sub-groups of adults under 40 years old sharing specific perspectives towards health, wellbeing and living with type 2 diabetes?
- Do these perspectives reveal specific barriers to and opportunities for better type 2 diabetes prevention and management and improved wellbeing?

For peer review only - http://bmjopen.bmj.com/site/about/guidelines.xhtml

Methods

Study design

The present study is part of the Cities Changing Diabetes protocol [11], which forms part of a wider global mixed methods approach [12]. This study follows the earlier 'Rule of Halves' analysis and delivers the 'Urban Diabetes Priority Assessment' [13] and consists of two components. The first component required participants to complete an online demographic questionnaire and computer based Q-sort, where participants prioritise a set of 64 statements according to their personal preference. These statements reflect eight social factors and cultural determinants of diabetes vulnerability [3]. The second component employed focus group discussions and individual interviews to facilitate in-depth exploration of the outcome of the Q-sort with selected participants [14].

Participants and recruitment

Adults aged between 18 and 40, with a confirmed type 2 diabetes diagnosis, and living in Greater Manchester were eligible to participate. The decision to focus recruitment on the 18-40 age group reflected findings from the Greater Manchester 'Rule of Halves' analysis. Participants were recruited through a combination of approaches: invitation by their GP or pharmacy; invitation via a consent-to-contact database (Help BEAT Diabetes); articles in Diabetes UK newsletters; enlisting community leaders within South Asian communities; and direct advertising on social media platforms (Facebook, Instagram, Twitter). Interested participants contacted researchers, and were eligible if they were over 18 years with diagnosed type 2 diabetes and lived in Greater Manchester. Participants were screened using a stratified sampling approach to ensure a diverse range of experiences, in regard to age, gender, ethnicity and socioeconomic status.

Data collection

After giving informed consent, eligible participants were granted access to our online data collection tool (Qsortware) [15]. Initially, participants completed a demographic survey (socioeconomic status, housing, employment, health insurance, long-term conditions, diabetes complications, height and weight). Next they undertook a statement sorting exercise ('Q-sort') to assess their perspectives on living with type 2 diabetes. Finally, a sample took part in interviews and focus groups.

Q-sort

The 64 statements had been synthesised from participant interviews in prior studies and represent a broad range of social and cultural factors relevant to health, well-being and type 2 diabetes [16]. The social factors were financial, time, resource, and geographic constraints and the cultural factors were traditions and conventions, health and illness, change and transition, self and other, and agency and choice [3].

In the sorting exercise, participants were instructed to rank statements based on their individual level of agreement or disagreement with each statement. In the first step (preliminary sort), participants were asked to sort statements into three "piles" (agree/disagree/neutral). In the second step (the final sort), participants added statements into a forced distribution grid (the Q-sort - see figure 1). We encouraged participants to add open-ended comments about each statement in a final step.

Figure 1. Example of a forced distribution grid (Watts & Stenner, 2012 p.17)

For peer review only - http://bmjopen.bmj.com/site/about/guidelines.xhtml

[see separate file]

Interviews & Focus Groups

The focus groups and interviews were led by an experienced qualitative health researcher (SC) with one or more other experienced researchers (AV, CP and RAA) in support, conducted remotely via secure video conferencing software (e.g. Zoom or MS Teams), digitally recorded (audio only) and professionally transcribed verbatim. Topic guides covered the journey to diagnosis, type 2 diabetes management, and the experiences of living with type 2 diabetes in Greater Manchester. We also explored areas where support was lacking and recommendations that could improve the experience of being a younger adult with type 2 diabetes. Focus Groups typically lasted 90 minutes and Interviews lasted 60 minutes.

Data Analysis

We used Zabala's 'qmethod' analysis package [17], run in R, to extract factors from the Q-sort and explore shared perspectives in our sample. As is customary in Q-methodology, the research team (AV, AP, SC, LB, CP, RA) discussed various solutions and used the 'factor array' (see appendix 1) to identify defining statements for each sub-group. 'Crib sheets' were used to extract the characteristics of each sub-group from 1) statements that ranked the highest in the factor compared to other factors; 2) statements that ranked the lowest in the factor compared to the other factors; 3) statements that ranked at -6 (very strongly disagree) ; and 4) statements that ranked at +6 (very strongly agree). The configuration of items and their interrelationships provided the basis for initial interpretations of participant perspectives for each sub-group.

Thematic coding in NVivo12 and rapid assessment procedure (RAP) sheets [18, 19] were used to provide an in-depth understanding of the issues that underpin the Q-sort and supplement our initial interpretations for each sub-group (see appendix 2). These were linked to the demographic data and the open-ended comments from the Q-sort, to provide a comprehensive overview.

Patient and public Involvement

Despite being part of our protocol, we were unable to recruit any younger adults living with type 2 diabetes as patient and public contributors for this study. This reflected the challenges of the COVID-19 pandemic and a general lack of engagement by this age group with formal health and community support services (e.g. Diabetes UK; Help BEAT Diabetes; local community champions).

For peer review only

For peer review only - http://bmjopen.bmj.com/site/about/guidelines.xhtml

Results

From 46 completions, 43 participants provided analysable data in the Q-sort. Of these, 17 (40%) participated in a focus group or interview. Table 1 shows a summary of participant characteristics.

Table 1. Summary of participants (n = 43)

Participant characteristic		
Age (years)	Mean	35.3
	Standard Deviation	4.4
Sex	Male	67%
	Female	33%
Body Mass Index (n=41)	Healthy weight (BMI 18.5 – 24.9)	12.2%
	Overweight (BMI 25 – 29.9)	7.3%
	Obese (BMI 30 – 39.9)	41.5%
	Severely obese (BMI over 40)	39.0%
Ethnicity	White	74.4%
	Asian/Asian British	20.9%
	Black/African/Caribbean/Black British	2.3%
	Multiple Ethnic Groups	2.3%
Household income (£)	<£30k	46.5%
	≥£30k	53.5%

We extracted 5 factors (representing 81% of participants) to create our sub-groups. Based upon their characteristics, we named these sub-groups 1) Stressed and calamity coping (n=13), 2) Financially disadvantaged and poorly supported (n=12), 3) Well-intentioned but not succeeding (n=5), 4) Withdrawn and worried (n=2), 5) Young and stigmatised (n=3). Holistic analysis of our qualitative data also identified some common issues across these sub-groups: overwhelming care obligations in those with young families; early or mid-career work pressures and stress; exclusion from social life; experiences with health professionals; and lack of specific skills and knowledge that could support them better. These are potentially more relevant to adults aged 18-40 and suggest a lack of personalisation within current health and support services.

Eight participants did not appear to fit any sub-group. These individuals still shared some common experiences with those in the 5 sub-groups and two participated in qualitative interviews. Their inclusion in our holistic analysis provides additional interpretation and contextualisation.

<u>Sub-group 1: 'Stressed and calamity coping' (n=13; qualitative interviews n=7)</u>

These participants were mostly female (average age 34). Despite initial challenges at diagnosis, they reported good awareness and some ability to manage their diabetes. They often ignored the onset of type 2 diabetes, being unclear about early symptoms.

> "I don't know how long I was diabetic for, I wouldn't be surprised if I've been diabetic for at least a couple of years before my diagnosis. Now it's one of those cases where you realise signs were there but they weren't as prominent" (a male ages 20-30 years old, BMI 51)

They expressed a genuine desire to 'turn things around' but managing other demands (including stress, work and dependants) often made it difficult. Self-described 'food addiction' was also a feature.

For peer review only - http://bmjopen.bmj.com/site/about/guidelines.xhtml

> "Yeah, because I think food addiction is very real but there is no recognition for it really anywhere." (a female ages 30-40 years old, BMI 43)

<u>Sub-group 2: 'Financially disadvantaged and poorly supported' (n=12; qualitative interviews n=4)</u>

Participants in this sub-group were mostly female (average age 36). They reported lacking skills, finances and support to better manage their type 2 diabetes and already have some complications. People were often 'pre-diabetic' for some time before being diagnosed with type 2 diabetes. Following diagnosis, they felt unsure about how to manage their symptoms and did not always know how to find further information.

> "But there was nothing, it was just like right, well, no potatoes, come off the coke, do more exercise, see you later". (a female ages 30-40 years old, BMI 50)

They would prefer more support with diet; although time, skills and money for healthy eating and exercise were barriers. They also perceive a clinical focus on medication, despite struggling with side-effects. They often felt embarrassed, self-conscious and judged by others.

> "I haven't really told everybody either. I haven't told my partner; he doesn't know. I've told my mum but I think it's the same, it's that you feel like people are going to judge you for having it and it's your fault because of the way that you eat." (a female ages 30-40 years old, BMI 40)

<u>Sub-group 3: 'Well-intentioned but not succeeding' (n=5; qualitative interviews n=3)</u>

These participants were mostly male (average age 37). They reported being proactive in managing their health and trying to balance their diagnosis within their wider circumstances and lifestyles. People regarded being overweight as 'socially acceptable'. Many have already developed associated complications. Often diagnosed as 'pre-diabetic' prior to full onset, they reported a family history of diabetes and expected a diagnosis sooner or later; although they were surprised to be diagnosed at such a young age.

> "It's my turn sooner or later it would be anyway." (a male ages 30-40 years old, BMI 29)

Family responsibilities were viewed as a positive motivation for change, and they felt that diet was more important than exercise in controlling type 2 diabetes. They like to ask a lot of questions and are quite optimistic about the future.

> "I think it's in moderation, and to be quite sensible with things, and what works for you. I've given up smoking, probably about five or six years too late, and again, because of my daughter, and priorities and things like that." (a male ages 30-40 years old, BMI 29).

<u>Sub-group 4: 'Withdrawn and worried' (n=2; qualitative interviews n=0)</u>

These participants were both male (average age 35). They reported being isolated with concerns about future health. They found type 2 diabetes scary and would have tried to avoid it, if they had known more about it earlier. They were unsure whom to trust with their healthcare and/or advice. They found it hard to learn about their type 2 diabetes and do not feel well equipped to take care of themselves. They feel their choices are limited, increasing anxiety about their health. They find it hard to socialise (because of diabetes) which increases feelings of loneliness. We were not able to directly engage with participants in this sub-group for a focus group or interview, so have not supplemented our initial interpretations.

For peer review only - http://bmjopen.bmj.com/site/about/guidelines.xhtml

Sub-group 5: 'Young and stigmatised' (n=3; qualitative interviews n=1)

Participants were a mix of female and male (average age 33).They reported being too young to worry too much about complications, although may have already experienced serious complications (e.g. acute kidney injury). They reported embarrassment at having type 2 diabetes so young, and found it hard to accept their diagnosis.

> "One of the doctors were like, you can go blind and you can…and I'm like yeah, I'm not going to go blind, I'm not 60 yet." (a female ages 20-30 years old, BMI 24)

They believe type 2 diabetes can be reversed, with diet and exercise, but it is hard without the right environment and support. They struggle to tolerate side-effects of medications, and choose non-adherence where these are felt not compatible with their age and lifestyle. There is some resentment towards health services.

> "I was like I can't even socialise, I can't go even go out. And especially when I was younger I was like I'm not doing this. So, I stopped taking the injections at that point." (a female ages 20-30 years old, BMI 24)

Common issues

Overwhelming care obligations in those with young families

The busy nature of peoples' lives often meant that, whatever their different situations, making the best choices was not easy. People across all sub-groups reported many barriers to lifestyle improvements. The combination of long working hours and childcare responsibilities made many people feel so tired at the end of the day that they would often reach for convenience foods. A lack of time, cooking skills or money were also reasons for less healthy choices. We saw signs of a gender split, with males largely citing family responsibilities as their motivation to 'do better', while females largely found these a barrier. Family life events and balancing responsibility for older and younger generations were a common source of stress.

Early or mid-career work pressures and stress

Many participants struggled to accept their diagnosis at such a young age. They often had other priorities, such as socialising, progressing their careers and getting on the housing 'ladder'. Long working hours made it difficult to access exercise classes or gyms at quieter and cheaper times. For some, working patterns and environments made it difficult to take regular breaks, eat healthily or ask for more support. The workplace was viewed as somewhere people feel they could be better supported. A lack of employer support made it more difficult to adopt good type 2 diabetes management.

Exclusion from social life

A number of our participants spoke about feeling embarrassed or ashamed of being diagnosed with type 2 diabetes, or admitting their diagnosis to others. They often experienced unpleasant side effects from medication (flatulence, diarrhoea etc) which could impact their social life, and being self-conscious about body size/shape could prevent access to exercise classes. Many expressed a desire to engage more with other people with type 2 diabetes, via support or exercise groups, suggesting they felt social support to be beneficial but lacking.

For peer review only - http://bmjopen.bmj.com/site/about/guidelines.xhtml

Experiences with health professionals

People shared a range of journeys to diagnosis, including family history, gestational diabetes, and pancreatic injury. Some had a period of 'pre-diabetes' prior to diagnosis. Those having a type 2 diabetes diagnosis at a younger age felt that health professionals did not always listen to them or provide appropriate services or support, suited to their needs. Medication as a primary treatment was not popular with participants, yet few alternatives were presented when common medications caused intolerable side-effects. This often led to refusal to take any prescribed medications. The perceived emphasis on services for older people with type 2 diabetes left many feeling abandoned or stigmatised. Previous negative experiences with health professionals could also influence current experiences, and contributed to feelings that younger people do not get as much support as older people. People in all sub-groups felt they had not received enough education about 'being healthy' or recognising the 'signs of type 2 diabetes' prior to onset. The absence of formal support increased use of and dependency on internet sources, self-help books and anecdotal advice, which may not always be appropriate. A desire to 'reverse' or manage their type 2 diabetes with food and exercise, in the first instance, was met with a lack of support from health professionals. Health services were regarded as good quality once people were advanced into the 'complications' phase of type 2 diabetes, but poor in prevention and early management. Provision was perceived as better in surrounding towns and worse in the city.

Lack of specific skills or knowledge that could support them better

Despite their younger age, participants in our sample had very high BMIs – over 80% reporting data were classed as obese (BMI over 30) or severely obese (BMI over 40). However, many reported not having the skills or knowledge to manage their health alone. For those with young children this was a cause of additional concern, as it meant their children would be left to witness and/or deal with any crisis (hypo) episodes, and they were also concerned their children may repeat their habits. A number of people we spoke to had parents or older relatives who already had type 2 diabetes. The current provision of pre-diabetes and type 2 diabetes services was not felt to provide the skills or knowledge to meet the different needs of younger people. Younger adults across the sub-groups noted how their finances and commitments and daily schedules are different to many older adults.

For peer review only - http://bmjopen.bmj.com/site/about/guidelines.xhtml

Discussion

This study aimed to understand the perspectives and experiences of younger adults living with type 2 diabetes in Greater Manchester. We found five sub-groups which illustrated the everyday challenges people face and the different strengths they may have to moderate these. These groups cut across socioeconomic factors such as household income, employment status, housing type, and residence ownership. We also found common challenges related to this age group.

Strengths and limitations

A key feature of a Q-study is the ability to reveal shared perspectives among a group of participants, and for those to be understood holistically [20]. The Q-sort requires a minimum of 35 participants to ensure statistical validity and a maximum of 64 (i.e. not exceeding the number of statements). We faced some challenges in recruiting younger adults living with type 2 diabetes as participants for this study, which thereby extended our recruitment period. This may reflect both the COVID-19 pandemic and a general lack of engagement by this age group with formal health services. To address this, we utilised multiple routes to optimise recruitment. In addition, the restrictions of the COVID-19 pandemic forced us to conduct all data collection via online platforms, which limited some from participating while providing opportunity to others. Zabala's 'qmethod' analysis package [17] allowed us to extract factors and thereby establish shared perspectives in our sample that met statistical significance, despite sometimes having smaller numbers within some sub-groups. Stratified sampling ensured we had a diverse range of experiences, with regard to age, gender, ethnicity and socioeconomic status, represented.

Comparison with the wider literature

Previous literature focusing on type 2 diabetes with a younger adult age group have revealed some concerning data on trends and issues. To our knowledge, this is the first Q-sort study in this population.

Browne et al [6] have previously noted that younger adults are more likely to be obese with sub-optimal glycaemic control and have a higher risk of developing at least one severe diabetes complication by their 40s. We found that people across all sub-groups had very high BMI and that poor glycaemic control was common. Reasons for this varied between the sub-groups, however the multiple pressures, unacceptability of side effects and a lack of appropriate support were all felt to contribute.

Kunasegaran et al [7] and Nanayakkara et al [8] have both highlighted the issue and need to understand better the barriers patients face in regard to medication adherence, the impact of this on glycaemic control and how this can be improved. Browne et al [6] have also suggested that people aged ≤ 45 years may require more intensive psychological and self-care support compared with those who develop type 2 diabetes aged ≥ 45 years. From our study, poor toleration of medications and their side-effects was a common issue raised by people across all sub-groups. Others suggested a preference for non-pharmaceutical approaches (diet, counselling and exercise) as a primary approach but found little support from statutory services, and some simply felt they 'still had time' before needing to submit to control through medication.

Tan et al [10] and Nanayakkara et al [9] have previously called for effective targeting of younger patients, earlier in their journey, as longer type 2 diabetes duration increases risk of complications. We found that many people, including those with a family history of type 2 diabetes, did not know how to recognise early symptoms, or could easily confuse these with other causes. Those with a

For peer review only - http://bmjopen.bmj.com/site/about/guidelines.xhtml

family history also believed it was to some extent inevitable they would develop type 2 diabetes, regardless of their habits, although only expected this when they were older. People without a family history were often ashamed and considered it their fault for developing type 2 diabetes, as they had not followed healthier lifestyles earlier. The lack of appropriate education, both in schools and in pre-diabetes courses, was felt to have contributed to this situation.

Walker et al [21] used a quantitative approach to examine the contribution of social determinants on glycaemic control in all adults (aged 18+) and found interventions that target self-efficacy and social support lead to improvements in glycaemic control, while clinical interventions can lower the burden of disease and address mutable social determinants. The overall age of people in our study was younger, however, we also found that greater social support and personalisation were proposed as potential solutions to common issues.

Implications of the study

Across our sub-groups, our findings suggest a perception of a lack of personalisation of diabetes support for those aged 18-40. People living with type 2 diabetes at a younger age want to be healthier, fitter, and more engaged in their own care. They frequently worried about their health problems and were anxious about the future. A lack of targeted programmes, that take their particular circumstances into account, make it harder to prioritise their type 2 diabetes care and achieve the necessary 'lifestyle modification' to prevent, delay or manage type 2 diabetes well.

The people we spoke to share some common experiences/problems, but they require different solutions. Younger people with type 2 diabetes need their GPs and care providers to recognise that they are not just individual patients, but are often parents and/or partners that live in complex and busy households. In addition, while much type 2 diabetes prevention and management is currently geared towards an older population, people who develop type 2 diabetes during their teens, twenties and thirties will ultimately need to manage this condition for longer, raising their risk of serious complications in the future. The condition is also heavily stigmatised and younger patients may suffer disproportionately from this.

Conclusion

In Greater Manchester, younger adults are under-diagnosed, less likely to receive appropriate care and less likely to achieve their HbA1c treatment targets compared with older age groups. We have explored the experiences of younger adults with type 2 diabetes, in Greater Manchester, and provide insights to different approaches that might help them avoid, delay onset or live well with type 2 diabetes and impact the long term trajectory of type 2 diabetes in this population. Within our sub-groups, we found particular patterns of lifestyle and social challenges that could inform services how to be better designed, to be sensitive to the needs and preferences of particular groups, including how these are advertised, delivered and assessed.

For peer review only - http://bmjopen.bmj.com/site/about/guidelines.xhtml

Acknowledgements
The authors would like to thank Diabetes UK and Help BEAT Diabetes, hosted by Research for the Future (RftF): see https://www.ncbi.nlm.nih.gov/pmc/articles/PMC6805518/

Author contributions
PB secured the funding. SC, AV, AP, CP, RAA, and LM conducted the study and AV, AP, SC, CP and RAA designed and performed the analysis. SC drafted the paper and PB, AV, CP, RAA and LM critically revised it. The corresponding author attests that all listed authors meet the relevant criteria and no other meeting these have been omitted. SC acts as the guarantor for this study.

Data availability statement
Data are available upon reasonable request. The dataset used is available on reasonable request from the corresponding author.

Ethics approval statement
This study was approved by the North-West - Greater Manchester Central Research Ethics Committee (REC reference: 21/NW/0030), HRA and Health and Care Research Wales (HCRW) IRAS project ID: 281050. Participants gave informed consent to participate in the study before taking part.

Funding
This research was funded by the National Institute for Health and Care Research Applied Research Collaboration Greater Manchester (NIHR ARC-GM) grant reference NIHR200174. This research originates from work undertaken in the Cities Changing Diabetes (CCD) programme for Greater Manchester in partnership with and supported by Health Innovation Manchester and Novo Nordisk. CCD was launched in 2014 by the Steno Diabetes Center Copenhagen, University College London, and Novo Nordisk.

Disclaimer
The views expressed in this publication are those of the author(s) and not necessarily those of the National Institute for Health and Care Research or the Department of Health and Social Care.

Competing interests
None declared.

References

1. Galicia-Garcia, U., Benito-Vicente, A., Jebari, S., et al. Pathophysiology of Type 2 Diabetes Mellitus, *Int. Journal of Molecular Sciences*, 2020, Vol. 21:6275, doi:10.3390/ijms21176275
2. Chatterjee, S., Khunti, K. and Davies, M.J. Type 2 diabetes, *The Lancet*, 2017, Vol. 389, Issue 10085: P2239-2251, https://www.thelancet.com/journals/lancet/article/PIIS0140-6736(17)30058-2/fulltext#%20 (accessed 10/06/222)
3. Cities Changing Diabetes. Urban Diabetes: understanding the challenges and opportunities, 2016, available at: https://www.citieschangingdiabetes.com/content/dam/nnsites/ccd/en/knowledge-hub/reports/pdfs/CCD_BB_FULL_BOOK_FOR_CN_DTP.pdf (accessed 13/06/2022)
4. Mason, T., Whittaker, W., Dumville, J., et al. Estimating the 'Rule of Halves' for Greater Manchester: Initial analysis of data, NIHR ARC GM, University of Manchester, 2020: Manchester
5. Health Innovation Manchester. Cities Changing Diabetes: Mapping the challenge of type 2 diabetes in Greater Manchester, 2022, available at:

For peer review only - http://bmjopen.bmj.com/site/about/guidelines.xhtml

https://healthinnovationmanchester.com/wp-content/uploads/2022/06/Cities-Changing-Diabetes-Manchester-report-final-June-2022.pdf (accessed 17/06/2022)

6. Browne, J. L., Nefs, G., Pouwer, F. et al. Depression, anxiety and self-care behaviours of young adults with Type 2 diabetes: results from the International Diabetes Management and Impact for Long-term Empowerment and Success (MILES) Study, *Diabetic Medicine*, 2014, Vol. 32:133–140, DOI: 10.1111/dme.12566
7. Kunasegaran, S., Beig, J., Khanolkar, M. et al. Adherence to medication, glycaemic control and hospital attendance in young adults with type 2 diabetes, *Internal Medicine Journal*, 2018, 48: 728–731 doi:10.1111/imj.13808
8. Nanayakkara, N., Pease, A.J., Ranasinha, S., et al. Younger people with Type 2 diabetes have poorer self-care practices compared with older people: results from the Australian National Diabetes Audit, *Diabetic Medicine*, 2018, Vol. 35 (8):1087-1095, DOI: 10.1111/dme.13660
9. Nanayakkara, N., Curtis, A.J., Heritier, S., et al. Impact of age at type 2 diabetes mellitus diagnosis on mortality and vascular complications: systematic review and meta-analyses, *Diabetologia*, 2020, 64 (2):275-287. doi: 10.1007/s00125-020-05319-w.
10. Tan, N.C., Barbier, S., Lim, W.Y. et al. 5-Year longitudinal study of determinants of glycemic control for multi-ethnic Asian patients with type 2 diabetes mellitus managed in primary care, *Diabetes research and clinical practice*, 2015, Vol. 110: 218-223
11. Cities Changing Diabetes. Urban Diabetes Priority Assessment, 2018, available at: https://www.citieschangingdiabetes.com/content/dam/nnsites/ccd/en/knowledge-hub/urban-diabetes-toolbox/pdfs/URBAN%20DIABETES%20PRIORITY%20ASSESSMENT_Intro%20Manual_Q22019_Screen.pdf (accessed 30/01/2022)
12. Napier, A.D., Nolan, J.J., Bagger, M., et al. Study protocol for the Cities Changing Diabetes programme: a global mixed-methods approach, *BMJ Open*, 2017, 7:e015240. doi: 10.1136/bmjopen-2016-015240
13. Cities Changing Diabetes. Urban Diabetes Toolbox, 2022, available at: https://www.citieschangingdiabetes.com/action-framework/urban-diabetes-toolbox.html (accessed 03/08/2022)
14. Volkmann, A.M., Hesseldal, L., Napier, D. et al. Urban Diabetes Priority Assessment, 2018, available at: https://www.citieschangingdiabetes.com/content/dam/nnsites/ccd/en/knowledge-hub/urban-diabetes-toolbox/pdfs/URBAN%20DIABETES%20PRIORITY%20ASSESSMENT_Intro%20Manual_Q22019_Screen.pdf (accessed 10/06/2022)
15. Pruneddu, A. and Zentner, M. QSortware, 2012, available at: www.qsortware.com.
16. Cities Changing Diabetes Bending The Curve On Urban Diabetes: New research approaches and innovative interventions for tackling diabetes in your city, 2017, available at: https://www.citieschangingdiabetes.com/content/dam/nnsites/ccd/en/knowledge-hub/reports/pdfs/14845_CCD17_BriefingBook_A4_09102017_LR.pdf (accessed 16/06/2022)
17. Zabala, A. Qmethod: A package to explore human perspectives using Q methodology, *R Journal*, 2014, 6 (2), 163-173. https://doi.org/10.32614/rj-2014-032 available at: https://www.repository.cam.ac.uk/handle/1810/248225 (accessed 05/08/2022)
18. Taylor, B., Henshall, C., Kenyon, S., et al. Can rapid approaches to qualitative analysis deliver timely, valid findings to clinical leaders? A mixed methods study comparing rapid and thematic analysis, *BMJ Open*, 2018, Vol. 8(10): e019993. doi:10.1136/bmjopen-2017-019993.

For peer review only - http://bmjopen.bmj.com/site/about/guidelines.xhtml

19. Vindrola-Padros, C., Chisnall, G., Cooper, S., et al. Carrying Out Rapid Qualitative Research During a Pandemic: Emerging Lessons From COVID-19, *Qualitative Health Research,* 2020, Vol. 30(14): 219-2204. doi.org/10.1177/1049732320951526.
20. Watts, S. and Stenner, P. Doing Q Methodological Research: Theory, Method and Interpretation, London, 2012: Sage Publications Ltd
21. Walker, R.J., Smalls, B.L. and Egede, L.E. Social determinants of health in adults with type 2 diabetes—Contribution of mutable and immutable factors, *Diabetes research and clinical practice*, 2015, Vol. 110: 193-201

[Appendix 1 see separate file]

[Appendix 2 see separate file]

For peer review only

CCD/SC/Figure 1

"Understanding the health impact of social and cultural influences on people aged 18-40 living with type 2 diabetes: a mixed-methods study"

Figure 1. Example of a forced distribution grid (Watts & Stenner, 2012 p.17)

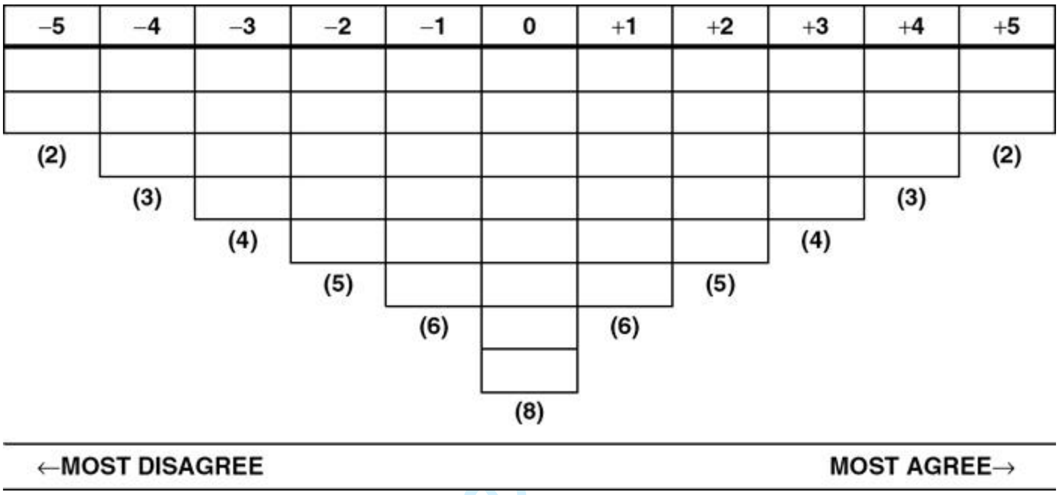

For peer review only

Appendix 1. GM factor array

	Factor 1	Factor 2	Factor 3	Factor 4	Factor 5
1 Diabetes is the least of my worries	-4	-4	0	-6	2
2 I worry about the cost of my diabetes	-3	3	-4	0	0
3 The way I make decisions about my healthcare is mostly dependent on how much money I have	-1	4	-2	3	-3
4 When it comes to food, I usually sacrifice quality for quantity	-1	-2	-4	5	6
5 Healthy foods are a luxury	-1	3	-1	1	1
6 Spending money on exercise classes or the gym is something I find difficult to do	1	5	-2	5	-1
7 Not having enough time makes people sick	1	2	-1	-4	-1
8 If I had more time, I'd exercise regularly	0	1	0	0	4
9 I just don't have the time to buy and cook healthy food	-4	1	-4	0	0
10 Diabetes support groups may be helpful, but I don't have time to participate	1	-1	2	6	-1
11 Taking care of others takes up most of my free time	0	5	-2	-2	2
12 I just don't have the skills to cook healthy meals	-3	-2	-5	1	3
13 For me, it's easy to learn about diabetes	4	0	3	-1	4
14 I think that diabetes or health-related support groups are pointless	-4	-3	0	-5	0
15 Where I live, fresh groceries are hard to get	-5	-3	-4	0	5
16 Living in the city is stressful and harmful to my health and wellbeing	-4	-1	1	-1	-5
17 I'd rather live elsewhere, but I have to live where I do because of work	-3	-4	4	2	-4
18 I am quite concerned about air pollution	0	0	0	3	-3
19 I think that where I live, it's not very safe to be out alone	-1	3	-5	-3	3
20 The climate where I live makes it really hard for me to want to walk or go somewhere by bicycle	-1	-1	-3	-1	-5
21 For me, it is important that I can get around by bike or on foot easily	0	0	-1	1	0
22 I like certain foods or dishes especially because they remind me of my childhood	1	1	1	4	-3
23 Cooking for those I love is a way of showing that I care for them	0	0	5	3	-1
24 When my family or my friends and I meet, we eat!	3	1	2	-1	-3
25 When I am by myself, I often eat food that is not very healthy	3	6	1	1	1
26 When I eat out together with friends, I usually end up eating unhealthy foods	6	4	3	-4	3
27 I often feel lonely	-5	2	2	6	2
28 To me, food is about more than just calorie intake	2	4	6	-1	3
29 Society puts a lot of pressure on women to be slim	5	5	-2	4	-3
30 I need my friends and social life to feel happy	4	0	-1	-2	-4
31 Being alone does not mean being lonely	4	2	3	0	-2
32 I tend to prioritise the needs of others over my own	3	6	0	1	-4
33 Men tend to be less interested in taking care of themselves than women	-2	-1	2	1	1
34 I have some health concerns that are more important to me than my diabetes	-2	0	1	-3	2
35 With today's medicine, diabetes is not a scary illness anymore	2	-2	3	-6	0
36 Diabetes is a disease of overweight people	-2	-5	2	-2	0
37 It is my fault that I now have diabetes	1	1	6	0	5
38 If I had known what having diabetes is like I would have tried harder to avoid becoming diabetic	2	3	0	3	-1
39 If I really had to, I could adjust to a healthy lifestyle	5	2	4	4	-2
40 I usually do what my doctor tells me to do	6	0	-1	-5	4
41 I avoid socialising because of my diabetes	-6	-3	-6	2	-1
42 I find it helpful to talk to other people about my diabetes	3	-2	-3	4	1
43 The modern world makes us ill	-1	1	3	1	-2
44 Feeling integrated with a community is important to me	1	-3	-1	3	1
45 Changes in my neighbourhood worry me	0	-1	0	0	4
46 I don't really trust health services or doctors	-6	-2	0	-5	0
47 Everybody just seems to be a bit bigger nowadays	1	0	5	0	2
48 My weight does not matter to me because I feel like a healthy person	-2	-6	-2	-3	5
49 Overweight people are often lazy	-2	-3	5	2	0
50 I am too embarrassed to go to the gym because of how I look	-1	2	0	-1	-5
51 Compared to others, I take good care of myself	0	-4	-3	-4	-2
52 I know who I can trust with my healthcare	2	0	-1	-2	1
53 I generally trust the government	0	-5	-6	-3	6
54 'Fitness freaks' have no joy in life	-3	-4	-3	2	2
55 I think that diabetes is inherited	2	1	1	-1	0
56 I think that diabetes is a death sentence	-5	-2	-2	2	-6
57 Most days, I feel good about my future	3	-1	-3	-2	3
58 At this point in my life, I feel like the choices I make are my own	5	4	4	-3	1
59 I get annoyed or upset with myself if I don't do enough for my health	2	3	1	5	-2
60 If I decide to be overweight or not exercise enough, that should be my choice	0	2	2	0	0
61 We owe it to society to be fit and healthy	0	0	1	-3	-4
62 Being unhealthy means you've given up on life	-3	-5	0	2	-1
63 Managing diabetes well is simply a matter of making the right choices	4	-1	4	-2	-2
64 My church is my rock	-2	-6	-5	0	-6
**NB: Distinguishing units are coloured**					

For peer review only

CCD/SC/Appendix 2

Appendix 2. Example RAP sheet

**Factor 1**	
**FGD/I participant(s):**<br>ID#<br>ID#	
**Q-sort comment(s):**<br>ID#<br>ID#<br>ID#<br>ID#	
**Analysis by:**	
**Diabetes journey**	
**Management**	
**GM lived experience**	
**Recommendations**	
**Notes (e.g. this interview a good source about x issue/standout quotations)**<br>ID#: “quote from transcript”<br>ID#: “quote from transcript”	

For peer review only

Consolidated criteria for reporting qualitative studies (COREQ): 32-item checklist

Please indicate in which section each item has been reported in your manuscript. If you do not feel an item applies to your manuscript, please enter N/A.

For further information about the COREQ guidelines, please see Tong *et al.*, 2017: https://doi.org/10.1093/intqhc/mzm042

No.	Item	Description	Section #
**Domain 1: Research team and reflexivity**			
Personal characteristics			
1.	Interviewer/facilitator	Which author/s conducted the interview or focus group?	p.6
2.	Credentials	What were the researcher's credentials? *E.g. PhD, MD*	p.1
3.	Occupation	What was their occupation at the time of the study?	p.1
4.	Gender	Was the researcher male or female?	N/A
5.	Experience and training	What experience or training did the researcher have?	p.6
Relationship with participants			
6.	Relationship established	Was a relationship established prior to study commencement?	p.5
7.	Participant knowledge of the interviewer	What did the participants know about the researcher? *E.g. Personal goals, reasons for doing the research*	N/A
8.	Interviewer characteristics	What characteristics were reported about the interviewer/facilitator? *E.g. Bias, assumptions, reasons and interests in the research topic*	N/A
**Domain 2: Study design**			
Theoretical framework			
9.	Methodological orientation and theory	What methodological orientation was stated to underpin the study? *E.g. grounded theory, discourse analysis, ethnography, phenomenology, content analysis*	p.5
Participant selection			
10.	Sampling	How were participants selected? *E.g. purposive, convenience, consecutive, snowball*	p.5
11.	Method of approach	How were participants approached? *E.g. face-to-face, telephone, mail, email*	p.5
12.	Sample size	How many participants were in the study?	p.7
13.	Non-participation	How many people refused to participate or dropped out? What were the reasons for this?	N/A
Setting			
14.	Setting of data collection	Where was the data collected? *E.g. home, clinic, workplace*	p.5-6
15.	Presence of non-participants	Was anyone else present besides the participants and researchers?	N/A

For peer review only

16.	Description of sample	What are the important characteristics of the sample? *E.g. demographic data, date*	p.5
Data collection			
17.	Interview guide	Were questions, prompts, guides provided by the authors? Was it pilot tested?	p.6
18.	Repeat interviews	Were repeat interviews carried out? If yes, how many?	N/A
19.	Audio/visual recording	Did the research use audio or visual recording to collect the data?	p.6
20.	Field notes	Were field notes made during and/or after the interview or focus group?	N/A
21.	Duration	What was the duration of the interviews or focus group?	p.6
22.	Data saturation	Was data saturation discussed?	N/A
23.	Transcripts returned	Were transcripts returned to participants for comment and/or correction?	N/A
**Domain 3: analysis and findings**			
Data analysis			
24.	Number of data coders	How many data coders coded the data?	p.13
25.	Description of the coding tree	Did authors provide a description of the coding tree?	N/A
26.	Derivation of themes	Were themes identified in advance or derived from the data?	p.7
27.	Software	What software, if applicable, was used to manage the data?	p.6
28.	Participant checking	Did participants provide feedback on the findings?	N/A
Reporting			
29.	Quotations presented	Were participant quotations presented to illustrate the themes / findings? Was each quotation identified? *E.g. Participant number*	p.7-9
30.	Data and findings consistent	Was there consistency between the data presented and the findings?	p.7-10
31.	Clarity of major themes	Were major themes clearly presented in the findings?	p.9-10
32.	Clarity of minor themes	Is there a description of diverse cases or discussion of minor themes?	p.11-12

When submitting your manuscript via the online submission form, please upload the completed checklist as a Figure/supplementary file.

If you would like this checklist to be included alongside your article, we ask that you upload the completed checklist to an online repository and include the guideline type, name of the repository, DOI and license in the *Data availability* section of your manuscript.

Developed from: Allison Tong, Peter Sainsbury, Jonathan Craig, Consolidated criteria for reporting qualitative research (COREQ): a 32-item checklist for interviews and focus groups, International Journal for Quality in Health Care, Volume 19, Issue 6, December 2007, Pages 349–357, https://doi.org/10.1093/intqhc/mzm042

BMJ Open

What are the perspectives of adults aged 18-40 living with type 2 diabetes in urban settings towards barriers and opportunities for better health and wellbeing: a mixed-methods study

Journal:	*BMJ Open*
Manuscript ID	bmjopen-2022-068765.R1
Article Type:	Original research
Date Submitted by the Author:	02-Mar-2023
Complete List of Authors:	Croke, Sarah; The University of Manchester,<br>Volkmann, Anna-Maria; University College London, Anthropology<br>Perry, Catherine ; The University of Manchester, Division of Population Health, Health Services Research and Primary Care<br>Atkinson, Ross; University of Manchester, Manchester, M13 9PL , Division of Nursing, Midwifery and Social Work, School of Health Sciences, Faculty of Biology, Medicine and Health;<br>Pruneddu, Alessio; Independent research consultant<br>Morris, Lydia; The University of Manchester, Division of Psychology and Mental Health, School of Health Sciences<br>Bower, Peter; University of Manchester, NPCRDC
**Primary Subject Heading**:	Diabetes and endocrinology
Secondary Subject Heading:	Health services research
Keywords:	PRIMARY CARE, QUALITATIVE RESEARCH, DIABETES & ENDOCRINOLOGY

SCHOLARONE™
Manuscripts

I, the Submitting Author has the right to grant and does grant on behalf of all authors of the Work (as defined in the below author licence), an exclusive licence and/or a non-exclusive licence for contributions from authors who are: i) UK Crown employees; ii) where BMJ has agreed a CC-BY licence shall apply, and/or iii) in accordance with the terms applicable for US Federal Government officers or employees acting as part of their official duties; on a worldwide, perpetual, irrevocable, royalty-free basis to BMJ Publishing Group Ltd ("BMJ") its licensees and where the relevant Journal is co-owned by BMJ to the co-owners of the Journal, to publish the Work in this journal and any other BMJ products and to exploit all rights, as set out in our licence.

The Submitting Author accepts and understands that any supply made under these terms is made by BMJ to the Submitting Author unless you are acting as an employee on behalf of your employer or a postgraduate student of an affiliated institution which is paying any applicable article publishing charge ("APC") for Open Access articles. Where the Submitting Author wishes to make the Work available on an Open Access basis (and intends to pay the relevant APC), the terms of reuse of such Open Access shall be governed by a Creative Commons licence – details of these licences and which Creative Commons licence will apply to this Work are set out in our licence referred to above.

Other than as permitted in any relevant BMJ Author's Self Archiving Policies, I confirm this Work has not been accepted for publication elsewhere, is not being considered for publication elsewhere and does not duplicate material already published. I confirm all authors consent to publication of this Work and authorise the granting of this licence.

For peer review only

CCD/SC/Manuscript_Final_(2)

- Title of the article

“What are the perspectives of adults aged 18-40 living with type 2 diabetes in urban settings towards barriers and opportunities for better health and wellbeing: a mixed-methods study”

- Full name, postal address and e-mail of the corresponding author

1. Dr Sarah Croke, PhD

Centre for Primary Care and Health Services Research, Division of Population Health, Health Services Research & Primary Care, School of Health Sciences, Faculty of Biology, Medicine and Health, University of Manchester, Suite 1 6th Floor Williamson Building, Oxford Road, Manchester, UK, M13 9PL. tel: 07932 011581 email: sarah.croke@manchester.ac.uk

- Full name, department, institution, city and country of all co-authors

2. Dr Anna-Maria Volkmann, PhD

Department of Anthropology, University College London, London, UK. email: a.volkmann@ucl.ac.uk

3. Dr Catherine Perry, PhD

NIHR Applied Research Collaboration Greater Manchester (NIHR ARC GM), Division of Population Health, Health Services Research & Primary Care/Division of Nursing, Midwifery & Social Work, School of Health Sciences, Faculty of Biology, Medicine and Health, University of Manchester, Manchester, UK. email: catherine.perry@manchester.ac.uk

4. Dr Ross A. Atkinson, PhD

NIHR Applied Research Collaboration Greater Manchester (NIHR ARC GM), Division of Nursing, Midwifery & Social Work, School of Health Sciences, Faculty of Biology, Medicine and Health, University of Manchester, Manchester, UK. email: ross.atkinson@manchester.ac.uk

5. Dr Alessio Pruneddu, PhD

Independent research consultant, Salford, UK. email: info@qsortouch.com

6. Dr Lydia Morris, PhD

Division of Psychology and Mental Health, School of Health Sciences, Faculty of Biology, Medicine and Health, University of Manchester, Manchester, UK. email: lydia.morris@manchester.ac.uk

7. Professor Peter Bower, PhD

NIHR Applied Research Collaboration Greater Manchester (NIHR ARC GM), Centre for Primary Care and Health Services Research, Division of Population Health, Health Services Research & Primary Care, School of Health Sciences, Faculty of Biology, Medicine and Health, University of Manchester, Manchester, UK. email: peter.bower@manchester.ac.uk

- Word count, excluding title page, abstract, references, figures and tables

4772 (excludes participant quotes as results).

For peer review only - http://bmjopen.bmj.com/site/about/guidelines.xhtml

CCD/SC/Manuscript_Final_(2)

"What are the perspectives of adults aged 18-40 living with type 2 diabetes in urban settings towards barriers and opportunities for better health and wellbeing: a mixed-methods study"

Sarah Croke, Anna-Maria Volkmann, Catherine Perry, Ross A. Atkinson, Alessio Pruneddu, Lydia Morris, and Peter Bower.

Abstract

Objectives: Delivered as part of the global assessment of diabetes in urban settings, this study explores different aspects of living in Greater Manchester for adults aged 18-40 with type 2 diabetes. Primary questions were: 1) can we identify sub-groups of adults under 40 years old sharing specific perspectives towards health, wellbeing and living with type 2 diabetes; and 2) do these perspectives reveal specific barriers to and opportunities for better type 2 diabetes prevention and management and improved wellbeing?

Design*:* The study employed a mixed method design with data collected through demographic questionnaires, Q-sort statement sorting exercises, focus groups discussions and individual interviews.

Participants*:* Those aged between 18 and 40, with a confirmed type 2 diabetes diagnosis, and living in Greater Manchester were eligible to participate. A total of 46 people completed the Q-sort exercise and 43 were included in the final analysis. Of those, 29 (67%) identified as female and 32 (75%) as white. Most common time since diagnosis was between five and ten years.

Results: The Q-sort analysis categorised 35 of the 43 participants (81%) into five sub-groups. Based on average statement sorts for each sub-group, perspectives were characterised as: 1) Stressed and calamity coping (n=13), 2) Financially disadvantaged and poorly supported (n=12), 3) Well-intentioned but not succeeding (n=5), 4) Withdrawn and worried (n=2), 5) Young and stigmatised (n=3). Holistic analysis of our qualitative data also identified some common issues across these sub-groups.

Conclusions: Adults under 40 with type 2 diabetes are not a homogenous group, but fall into five identifiable sub-groups. They also experience issues specific to this age group that make it particularly difficult for them to focus on their own health. More tailored support could help them to make the necessary lifestyle changes and manage their type 2 diabetes better.

For peer review only - http://bmjopen.bmj.com/site/about/guidelines.xhtml

Article Summary

Strengths and limitations of this study

- A key feature of a Q-study is the ability to reveal shared perspectives among a group of participants, and for those to be understood holistically.
- The Q-sort requires a minimum of 35 participants to ensure statistical validity and a maximum of 64 (i.e. not exceeding the number of statements).
- The challenge to recruit younger adults living with type 2 diabetes as participants for this study were addressed by utilising multiple routes, including social media.
- Restrictions due to the COVID-19 pandemic forced us to conduct all data collection via online platforms; this limited some from participating while providing opportunity to others.
- Zabala's 'qmethod' analysis package allowed us to extract factors meeting statistical significance (high loading score on a single factor with $p<0.05$), and thereby establish shared perspectives in our sample despite sometimes having smaller numbers within some sub-groups.

For peer review only

For peer review only - http://bmjopen.bmj.com/site/about/guidelines.xhtml

Introduction

Type 2 diabetes is one of the most common metabolic disorders worldwide [1] and leads to complications that cause both psychological and physical distress and put a huge burden on healthcare systems [2]. One response to this, in recent years, has been the Cities Changing Diabetes programme, a programme advocating for urgent action against type 2 diabetes on a global scale. Launched in 2015, via a partnership between Novo Nordisk, University College London and Steno Diabetes Center, and implemented by City Partners, it aims to map the challenge, share solutions and act on the growing impact of type 2 diabetes in some of the world's great cities [3]. In 2019, Greater Manchester, UK became a City Partner.

A report conducted as part of this programme using the 'Rule of Halves' approach [4, 5] identified patterns of diagnosis and treatment in Greater Manchester and indicated that type 2 diabetes in the area is under-diagnosed in adults under 40 years of age. This also showed that, where type 2 diabetes was diagnosed, those under 40 seem less likely to receive appropriate care and achieve HbA1c treatment targets when compared with older age groups. Building on this, we conducted the Urban Diabetes Priority Assessment to explore shared priorities, attitudes, and points of view among local people with type 2 diabetes. Shared perspectives are important for informing future interventions and policies, while also helping to strengthen the global Cities Changing Diabetes research platform for understanding the social-cultural drivers of Diabetes [6].

Previous research suggests that adults aged 18-40 with type 2 diabetes have higher physical morbidity and mortality than other type 2 diabetes sub-groups and poorer outcomes than their older counterparts [7]. People in this group are also more likely to be obese with sub-optimal glycaemic control and have a high risk of developing at least one severe diabetes complication by the time they are in their 40s, reducing life expectancy by 15 years [7]. A further concern is poor adherence to medication [8, 9]. Being a younger age at diagnosis is also associated with higher risk of mortality and vascular disease [10], while people may require more intensive psychological and self-care support compared with those who develop type 2 diabetes later [7]. It is suggested that targeting younger patients, earlier in their trajectory, can present a unique opportunity to improve longer term outcomes [11].

We aimed to explore the following questions for adults under 40 with type 2 diabetes, living in Greater Manchester:

- Can we identify sub-groups of adults under 40 years old sharing specific perspectives towards health, wellbeing and living with type 2 diabetes?
- Do these perspectives reveal specific barriers to and opportunities for better type 2 diabetes prevention and management and improved wellbeing?

For peer review only - http://bmjopen.bmj.com/site/about/guidelines.xhtml

Methods

Study design

The present study is part of the Cities Changing Diabetes protocol [12], which forms part of a wider global mixed methods approach [13]. This study draws on the principles of Q-methodology, a technique increasingly used by researchers to explore health-related decision-making and behaviours via the systematic and scientific study of subjective viewpoints. The aim of a Q-study is to extract as many world-views as possible, obtaining perspectives and point of views from highly informed individuals rather than from a sample representative of the general population, and employs a mixture of data collection and analyses to obtain insights [14]. It follows the earlier 'Rule of Halves' analysis and delivers the 'Urban Diabetes Priority Assessment' [15] which consists of two components.

The first component required participants to complete an online demographic questionnaire and computer based Q-sort, where participants prioritise a set of 64 statements according to their personal preference. The statements in the Q-sort reflect eight social factors and cultural determinants of diabetes vulnerability and were generated from prior Cities Changing Diabetes Vulnerability Assessment studies conducted in five global cities: Copenhagen, Mexico City, Houston, Shanghai and Tianjin. Across those sites, 746 individual assessments were carried out by AMV and University College London (UCL) colleagues. Each assessment lasted on average two hours and centred around a specific interview guide which had been translated and locally-adapted. The assessments also captured demographic data via a survey as well as ethnographic observation data [13]. Examples of statements include: "Healthy foods are a luxury", "For me, it's easy to learn about diabetes", "I tend to prioritise the needs of others over my own", and "I think that diabetes is a death sentence" (full list in appendix 1). The local research teams as well as the global team (UCL) shared consensus that they adequately reflected the lived experience of the participants in the Cities Changing Diabetes Vulnerability Assessment. The statement set was first validated as part of the Cities Changing Diabetes Vancouver Q-Sort study, and further validated as part of the Cities Changing Diabetes Moscow Q-sort Study.

The second component employed focus group discussions and individual interviews to facilitate in-depth exploration of the outcome of the Q-sort with selected participants [6].

Participants and recruitment

Adults aged between 18 and 40, with a confirmed type 2 diabetes diagnosis, and living in Greater Manchester were eligible to participate. The decision to focus recruitment on the 18-40 age group reflected findings from the Greater Manchester 'Rule of Halves' analysis. Participants were recruited through a combination of approaches: invitation by their GP or pharmacy; invitation via a consent-to-contact database (Help BEAT Diabetes); articles in Diabetes UK newsletters; enlisting community leaders within South Asian communities; and direct advertising on social media platforms (Facebook, Instagram, Twitter). Interested participants contacted researchers whereupon they were screened using a stratified sampling approach to ensure a diverse range of experiences, in regard to age, gender, ethnicity and socioeconomic status.

Data collection

For peer review only - http://bmjopen.bmj.com/site/about/guidelines.xhtml

After giving informed consent, eligible participants were granted access to our online data collection tool (Qsortware) [16]. Initially, participants completed a demographic survey (socioeconomic status, housing, employment, health insurance, long-term conditions, diabetes complications, height and weight). Next they undertook a statement sorting exercise (‘Q-sort’) to assess their perspectives on living with type 2 diabetes. Finally, those who had consented to be contacted were invited for stage 2 interviews and focus groups. Participants who agreed joined others to explore the results from the computer-based data collection, adding depth and detail to the statistical data, following a Cities Changing Diabetes-specific interview protocol [14].

Q-sort

The 64 statements had been synthesised from participant interviews in prior studies and represent a broad range of social and cultural factors relevant to health, well-being and type 2 diabetes [13, 14]. The social factors were financial, time, resource, and geographic constraints and the cultural factors were traditions and conventions, health and illness, change and transition, self and other, and agency and choice [3].

In the sorting exercise, participants were instructed to rank statements based on their individual level of agreement or disagreement with each statement. In the first step (preliminary sort), participants were asked to sort statements into three “piles” (agree/disagree/neutral). In the second step (the final sort), participants added statements into a forced distribution grid (the Q-sort - see figure 1). We encouraged participants to add open-ended comments about each statement in a final step.

Figure 1. Example of a forced distribution grid (Watts & Stenner, 2012 p.17)

[see separate file]

Interviews & Focus Groups

The interviews and focus groups were led by an experienced qualitative health researcher (SC) with one or more other experienced researchers (AMV, CP and RAA) in support, conducted remotely via secure video conferencing software (e.g. Zoom or MS Teams), digitally recorded (audio only) and professionally transcribed verbatim. Topic guides covered the journey to diagnosis, type 2 diabetes management, and the experiences of living with type 2 diabetes in Greater Manchester (see appendix 2). We also explored areas where support was lacking and recommendations that could improve the experience of being a younger adult with type 2 diabetes. Focus Groups typically lasted 90 minutes and Interviews lasted 60 minutes.

Data Analysis

We used Zabala’s ‘qmethod’ analysis package [17], run in R, to extract factors from the Q-sort and explore shared perspectives in our sample. To be assigned to a factor, participants needed a high loading score on a single factor, with $p<.05$, and for this squared score to be higher than the squared sum of the scores obtained in all the other factors. Factors were described by the agreement, disagreement and neutrality expressed for each statement, both when compared to the other subgroup decision on that statement and in relation to the agreement/disagreement of the other statements of the Q-set, since there was a forced choice distribution involved. As is customary in Q-methodology, the research team (AMV, AP, SC, CP, RAA) discussed various solutions and used the ‘factor array’ (see appendix 1) to identify defining statements for each sub-group. A series of T-tests identified those statements that each subgroup sorted out differently, therefore helping the

For peer review only - http://bmjopen.bmj.com/site/about/guidelines.xhtml

interpretation. However, even those statements considered 'consensus' were still relevant, since they informed about issues/ barriers universally perceived as significant by each perspective. 'Crib sheets' were used to extract the characteristics of each sub-group from 1) statements that ranked the highest in the factor compared to other factors; 2) statements that ranked the lowest in the factor compared to the other factors; 3) statements that ranked at -6 (very strongly disagree) ; and 4) statements that ranked at +6 (very strongly agree). Interpretation of the factors began by taking together all the statements in the 'strongly agree' area and those in the 'strongly disagree' area, especially when this configuration is in stark contrast with the other sub-groups. The configuration of items and their interrelationships provided the basis for initial interpretations of participant perspectives for each sub-group.

Thematic coding in NVivo12 and rapid assessment procedure (RAP) sheets [18, 19] were used to provide further, in-depth understanding of the issues that underpin the Q-sort and to supplement initial interpretations for each sub-group (see appendix 3). These were synthesised with the demographic data and the open-ended comments from the Q-sort, to provide a comprehensive overview.

Patient and public Involvement

There was no formal PPI involvement in the design or planning of the study. The broad methods used and reported here are part of an established Cities Changing Diabetes protocol [12] with little option to make changes to the methods. Our project was embedded in a wider project that had stakeholder involvement as part of the project steering group, and we attempted to recruit younger adults living with type 2 diabetes as patient and public contributors to advise on delivery and dissemination of this study, but were unsuccessful in the timelines. This reflected the challenges of the COVID-19 pandemic and a general lack of engagement by this age group with formal health and community support services (e.g. Diabetes UK; Help BEAT Diabetes; local community champions).

For peer review only - http://bmjopen.bmj.com/site/about/guidelines.xhtml

Results

From 46 completions, 43 participants provided analysable data in the Q-sort. Of these, 17 (40%) participated in a focus group or interview. Table 1 shows a summary of participant characteristics.

Table 1. Summary of participants (n = 43)

Participant characteristic		
Age (years)	Mean	35.3
	Standard Deviation	4.4
Sex	Male	67%
	Female	33%
Body Mass Index (n=41)	Healthy weight (BMI 18.5 – 24.9)	12.2%
	Overweight (BMI 25 – 29.9)	7.3%
	Obese (BMI 30 – 39.9)	41.5%
	Severely obese (BMI over 40)	39.0%
Ethnicity	White	74.4%
	Asian/Asian British	20.9%
	Black/African/Caribbean/Black British	2.3%
	Multiple Ethnic Groups	2.3%
Household income (£)	<£30k	46.5%
	≥£30k	53.5%

We extracted five factors (representing 81% of participants) to create our sub-groups. Determining the best factor solution resulted from several discussions of the many variables involved, the complexity of the topic, and after excluding every factor solution with factors based on a single individual. We considered the total variance explained, the correlation between factors, the eigenvalues, and reliability coefficients for each factor (Humphrey's rule). Individual Q-sorts were assigned to a factor according to specific criteria set by 'q-method', the library from R we used for data analysis. This activity, called 'flagging', ensured each factor was highly distinguishable from the other ones extracted. All assigned participants achieved a high loading score on a single factor with $p<0.05$ and with the squared score higher than the squared sum of the scores obtained in all the other factors. We opted for a five factor solution since the variance explained was maximised in a stable statistical structure.

Based upon their characteristics, we named these sub-groups 1) Stressed and calamity coping (n=13), 2) Financially disadvantaged and poorly supported (n=12), 3) Well-intentioned but not succeeding (n=5), 4) Withdrawn and worried (n=2), 5) Young and stigmatised (n=3). These interpretations reflect the key viewpoints captured by each study factor created from their relative and distinguishing statement rankings and demographic information, illustrated by the interview and focus group data [20]. They are woven and presented in a *narrative* style to present a seamless account of each sub-group's holistic viewpoint.

Holistic analysis of our qualitative data also identified some common issues across these five identifiable sub-groups: overwhelming care obligations in those with young families; early or mid-career work pressures and stress; exclusion from social life; experiences with health professionals; and lack of specific skills and knowledge that could support them better. Some of these may be more relevant to adults aged 18-40 than other age groups and suggest a gap within current health and support services. Ethnicity had no clear influence on participant fit to sub-groups. Household

For peer review only - http://bmjopen.bmj.com/site/about/guidelines.xhtml

income showed potential influence in two of the sub-groups: in sub-group 3 almost all participants reported incomes over £30k, while in sub-group 4 all participants reported incomes under £30k, a figure which is broadly in line with the current UK median household income [20]. Eight participants did not appear to fit any sub-group. These individuals still shared some common experiences with those in the five sub-groups, including observations regarding ethnicity and household income, and two participated in qualitative interviews. Their inclusion in our holistic analysis provides additional interpretation and contextualisation.

Sub-group 1: 'Stressed and calamity coping' (n=13; qualitative interviews n=7)

These participants were mostly female (average age 34). Ten identified as white and three identified as Asian/Asian British. Their key viewpoint is that friends and family are very important. Despite initial challenges at diagnosis, they report having good awareness and some ability to manage their diabetes. They often ignore the onset of type 2 diabetes, and can be unclear about early symptoms.

> "I don't know how long I was diabetic for, I wouldn't be surprised if I've been diabetic for at least a couple of years before my diagnosis. Now it's one of those cases where you realise signs were there but they weren't as prominent" (a male ages 20-30 years old, BMI 51)

They expressed a genuine desire to 'turn things around' but managing other demands (including stress, work and dependants) often made it difficult. Self-described 'food addiction' was also a feature.

> "Yeah, because I think food addiction is very real but there is no recognition for it really anywhere." (a female ages 30-40 years old, BMI 43)

Sub-group 2: 'Financially disadvantaged and poorly supported' (n=12; qualitative interviews n=4)

Participants in this sub-group were mostly female (average age 36). Ten identified as white, one identified as Asian/Asian British and one identified as Black/African/Caribbean/Black British. Their key viewpoint is they prioritise the needs of others, at the expense of themselves. They report lacking skills, finances and support to better manage their type 2 diabetes and may already have some complications. People are often 'pre-diabetic' for some time before being diagnosed with type 2 diabetes. Following diagnosis, they can feel unsure about how to manage their symptoms and do not always know how to find further information.

> "But there was nothing, it was just like right, well, no potatoes, come off the coke, do more exercise, see you later". (a female ages 30-40 years old, BMI 50)

They would like more support with diet; although time, skills and money for healthy eating and exercise are barriers. They also perceive a clinical focus on medication, despite struggling with side-effects. They will often felt embarrassed, self-conscious and judged by others.

> "I haven't really told everybody either. I haven't told my partner; he doesn't know. I've told my mum but I think it's the same, it's that you feel like people are going to judge you for having it and it's your fault because of the way that you eat." (a female ages 30-40 years old, BMI 40)

Sub-group 3: 'Well-intentioned but not succeeding' (n=5; qualitative interviews n=3)

These participants were mostly male (average age 37). Three identified as white, one identified as Asian/Asian British and one identified as multiple ethnic groups. Their key viewpoint is they feel responsible for having diabetes, and so are responsible for managing it. They report being proactive in managing their health and trying to balance their diagnosis within their wider circumstances and

For peer review only - http://bmjopen.bmj.com/site/about/guidelines.xhtml

lifestyles. Some regard being overweight as 'socially acceptable' and can have already developed associated complications. Often diagnosed as 'pre-diabetic' prior to full onset, they may report a family history of diabetes and expect a diagnosis sooner or later; although they are surprised to be diagnosed at such a young age.

> "It's my turn sooner or later it would be anyway." (a male ages 30-40 years old, BMI 29)

Family responsibilities are viewed as a positive motivation for change, and they feel that diet is more important than exercise in controlling type 2 diabetes. They like to ask a lot of questions and are quite optimistic about the future.

> "I think it's in moderation, and to be quite sensible with things, and what works for you. I've given up smoking, probably about five or six years too late, and again, because of my daughter, and priorities and things like that." (a male ages 30-40 years old, BMI 29).

Sub-group 4: 'Withdrawn and worried' (n=2; qualitative interviews n=0)

These participants were both male (average age 35). One identified as white and one identified as Asian/Asian British. Their key viewpoint is being isolated with concerns about future health. They find type 2 diabetes scary and say they would have tried to avoid it, if they had known more about it earlier. They are unsure whom to trust with their healthcare and/or advice. They find it hard to learn about their type 2 diabetes and do not feel well equipped to take care of themselves. They feel their choices are limited, increasing anxiety about their health. They find it hard to socialise (because of diabetes) which increases feelings of loneliness.

In the absence of interview and focus group data, this interpretation and identification of key viewpoint relies solely on their relative and distinguishing statement rankings, plus demographic information.

Sub-group 5: 'Young and stigmatised' (n=3; qualitative interviews n=1)

Participants were a mix of female and male (average age 33). Two identified as white and one identified as Asian/Asian British. Their key viewpoint is there is more to life than diabetes. They report being too young to worry too much about complications, although may have already experienced serious complications (e.g. acute kidney injury). They feel embarrassment about having type 2 diabetes so young, and find it hard to accept their diagnosis.

> "One of the doctors were like, you can go blind and you can…and I'm like yeah, I'm not going to go blind, I'm not 60 yet." (a female ages 20-30 years old, BMI 24)

They believe type 2 diabetes can be reversed, with diet and exercise, but it is hard without the right environment and support. They struggle to tolerate side-effects of medications, and choose non-adherence where they feel these are not compatible with their age and lifestyle. There is some resentment towards health services.

> "I was like I can't even socialise, I can't go even go out. And especially when I was younger I was like I'm not doing this. So, I stopped taking the injections at that point." (a female ages 20-30 years old, BMI 24)

Common issues

For peer review only - http://bmjopen.bmj.com/site/about/guidelines.xhtml

In addition to identifying sub-groups who share specific perspectives towards health, wellbeing and living with type 2 diabetes, we also identified common issues across the sub-groups that create barriers to better type 2 diabetes prevention, management and improved wellbeing.

Overwhelming care obligations in those with young families

We learned the busy nature of peoples' lives often meant that, whatever their different situations, making the best choices was not easy. People across all sub-groups reported many barriers to lifestyle improvements. The combination of long working hours and childcare responsibilities made many people feel so tired at the end of the day that they would often reach for convenience foods. A lack of time, cooking skills or money were also reasons for less healthy choices. We saw signs of a gender split, with males largely citing family responsibilities as their motivation to 'do better', while females largely found these a barrier. Family life events and balancing responsibility for older and younger generations were a common source of stress.

Early or mid-career work pressures and stress

Many participants told us they struggled to accept their diagnosis at such a young age. They often had other priorities, such as socialising, progressing their careers and getting on the housing 'ladder'. Long working hours made it difficult to access exercise classes or gyms at quieter and cheaper times. For some, working patterns and environments made it difficult to take regular breaks, eat healthily or ask for more support. The workplace was viewed as somewhere people feel they could be better supported. A lack of employer support made it more difficult to adopt good type 2 diabetes management.

Exclusion from social life

A number of our participants spoke about feeling embarrassed or ashamed of being diagnosed with type 2 diabetes, or admitting their diagnosis to others. They often experienced unpleasant side effects from medication (flatulence, diarrhoea etc) which could impact their social life, and being self-conscious about body size/shape could prevent access to exercise classes. Many expressed a desire to engage more with other people with type 2 diabetes, via support or exercise groups, suggesting they felt social support to be beneficial but lacking.

Experiences with health professionals

People shared with us a range of journeys to diagnosis, including family history, gestational diabetes, and pancreatic injury. Some had a period of 'pre-diabetes' prior to diagnosis. Those having a type 2 diabetes diagnosis at a younger age felt that health professionals did not always listen to them or provide appropriate services or support, suited to their needs. Medication as a primary treatment was not popular with participants, yet few alternatives were presented when common medications caused intolerable side-effects. This often led to refusal to take any prescribed medications. The perceived emphasis on services for older people with type 2 diabetes left many feeling abandoned or stigmatised. Previous negative experiences with health professionals could also influence current experiences, and contributed to feelings that younger people do not get as much support as older people. People in all sub-groups felt they had not received enough education about 'being healthy' or recognising the 'signs of type 2 diabetes' prior to onset. The absence of formal support increased use of and dependency on internet sources, self-help books and anecdotal advice, which may not always be appropriate. A desire to 'reverse' or manage their type 2 diabetes with food and exercise, in the first instance, was met with a lack of support from health professionals. Health services were regarded as good quality once people were advanced into the 'complications' phase of type 2

For peer review only - http://bmjopen.bmj.com/site/about/guidelines.xhtml

diabetes, but poor in prevention and early management. Provision was perceived as better in surrounding towns and worse in the city.

Lack of specific skills or knowledge that could support them better

We found that, despite their younger age, participants in our sample often had very high BMIs – over 80% reporting data were classed as obese (BMI over 30) or severely obese (BMI over 40). However, many reported not having the skills or knowledge to manage their health alone. For those with young children this was a cause of additional concern, as it meant their children would be left to witness and/or deal with any crisis (hypo) episodes, and they were also concerned their children may repeat their habits. A number of people we spoke to had parents or older relatives who already had type 2 diabetes. The current provision of pre-diabetes and type 2 diabetes services was not felt to provide the skills or knowledge to meet the different needs of younger people. Younger adults across the sub-groups noted how their finances and commitments and daily schedules are different to many older adults.

For peer review only

Discussion

This study aimed to identify and understand the perspectives and experiences of younger adults living with type 2 diabetes in Greater Manchester. We found five sub-groups which illustrated the everyday challenges people face and the different strengths they may have to moderate these. These sub-groups cut across socioeconomic factors such as household income, employment status, ethnicity, housing type, and residence ownership. We also found common challenges related to this age group.

Strengths and limitations

A key feature of a Q-study is the ability to reveal shared perspectives among a group of participants, and for those to be understood holistically [21]. The Q-sort requires a minimum of 35 participants to ensure statistical validity and a maximum of 64 (i.e. not exceeding the number of statements). We faced some challenges in recruiting younger adults living with type 2 diabetes as participants for this study, which thereby extended our recruitment period. This may reflect both the COVID-19 pandemic and a general lack of engagement by this age group with formal health services. To address this, we utilised multiple routes to optimise recruitment. In addition, the restrictions of the COVID-19 pandemic forced us to conduct all data collection via online platforms, which limited some from participating while providing opportunity to others. Zabala's 'qmethod' analysis package [17] allowed us to extract factors meeting statistical significance (high loading score on a single factor with $p<0.05$), and thereby establish shared perspectives in our sample, despite sometimes having smaller numbers within some sub-groups. Stratified sampling ensured we had a diverse range of experiences, with regard to age, gender, ethnicity and socioeconomic status, represented.

Comparison with the wider literature

Previous literature focusing on type 2 diabetes with a younger adult age group have revealed some concerning data on trends and issues. To our knowledge, this is the first Q-sort study in this population.

Browne et al [7] have previously noted that younger adults are more likely to be obese with sub-optimal glycaemic control and have a higher risk of developing at least one severe diabetes complication by their 40s. We found that people across all sub-groups had very high BMI and that self-reported poor glycaemic control was common. Reasons for this varied between the sub-groups, however the multiple pressures, unacceptability of side effects, and a lack of appropriate support were all felt to contribute.

Kunasegaran et al [8] and Nanayakkara et al [9] have both highlighted the issue and need to understand better the barriers patients face in regard to medication adherence, the impact of this on glycaemic control and how this can be improved. Browne et al [7] have also suggested that people aged ≤ 45 years may require more intensive psychological and self-care support compared with those who develop type 2 diabetes aged ≥ 45 years. From our study, poor toleration of medications and their side-effects was a common issue raised by people across all sub-groups. Others suggested a preference for non-pharmaceutical approaches (diet, counselling and exercise) as a primary approach but found little support from statutory services, and some simply felt they 'still had time' before needing to submit to control through medication.

Tan et al [11] and Nanayakkara et al [10] have previously called for effective targeting of younger patients, earlier in their journey, as longer type 2 diabetes duration increases risk of complications. We found that many people, including those with a family history of type 2 diabetes, did not know

For peer review only - http://bmjopen.bmj.com/site/about/guidelines.xhtml

how to recognise early symptoms, or could easily confuse these with other causes. Those with a family history also believed it was to some extent inevitable they would develop type 2 diabetes, regardless of their habits, although only expected this when they were older. People without a family history were often ashamed and considered it their fault for developing type 2 diabetes, as they had not followed healthier lifestyles earlier. The lack of appropriate education, both in schools and in pre-diabetes courses, was felt to have contributed to this situation.

Walker et al [22] used a quantitative approach to examine the contribution of social determinants on glycaemic control in all adults (aged 18+) and found interventions that target self-efficacy and social support lead to improvements in glycaemic control, while clinical interventions can lower the burden of disease and address mutable social determinants. The overall age of people in our study was younger, however, we also found that greater social support and personalisation were proposed as potential solutions to common issues.

Implications of the study

Across our sub-groups, our findings suggest a perception of a lack of personalisation of diabetes support for those aged 18-40. People living with type 2 diabetes at a younger age want to be healthier, fitter, and more engaged in their own care. They frequently worried about their health problems and were anxious about the future. A lack of targeted programmes, that take their particular circumstances into account, make it harder to prioritise their type 2 diabetes care and achieve the necessary 'lifestyle modification' to prevent, delay or manage type 2 diabetes well.

The people we spoke to shared some common experiences/problems, but they each appeared to require different solutions. Younger people with type 2 diabetes need their GPs and care providers to recognise that they are not just individual patients, but are often parents and/or partners that live in complex and busy households. In addition, while much type 2 diabetes prevention and management is currently structured towards an older population, people who develop type 2 diabetes during their teens, twenties and thirties will ultimately need to manage this condition for longer, raising their risk of serious complications in the future. The condition is also heavily stigmatised and younger patients may suffer disproportionately from this.

Conclusion

The Rule of Halves study that informed our work, as part of the Cities Changing Diabetes protocol, suggested that in Greater Manchester younger adults are under-diagnosed, less likely to receive appropriate care and less likely to achieve their HbA1c treatment targets compared with older age groups. Using Q-methodology, we have explored the experiences of younger adults with type 2 diabetes, in Greater Manchester, identifying discrete sub-groups within this population and providing insights to different approaches that might help them avoid, delay onset or live well with type 2 diabetes. We also identify some common issues across the sub-groups that may impact their long term trajectory of type 2 diabetes and found particular patterns of lifestyle and social challenges that could inform services how to be better designed, to be sensitive to the needs and preferences of particular groups, including how these are advertised, delivered and assessed.

For peer review only - http://bmjopen.bmj.com/site/about/guidelines.xhtml

Acknowledgements
The authors would like to thank Diabetes UK and Help BEAT Diabetes, hosted by Research for the Future (RftF): see https://www.ncbi.nlm.nih.gov/pmc/articles/PMC6805518/

Author contributions
PB secured the funding. SC, AMV, AP, CP, RAA, and LM conducted the study and AMV, AP, SC, CP and RAA designed and performed the analysis. SC drafted the paper and PB, AMV, CP, RAA, AP and LM critically revised it. The corresponding author attests that all listed authors meet the relevant criteria and no other meeting these have been omitted. SC acts as the guarantor for this study.

Data availability statement
Data are available upon reasonable request. The dataset used is available on reasonable request from the corresponding author.

Ethics approval statement
This study was approved by the North-West - Greater Manchester Central Research Ethics Committee (REC reference: 21/NW/0030), HRA and Health and Care Research Wales (HCRW) IRAS project ID: 281050. Participants gave informed consent to participate in the study before taking part.

Funding
This research was funded by the National Institute for Health and Care Research Applied Research Collaboration Greater Manchester (NIHR ARC-GM) grant reference NIHR200174. This research originates from work undertaken in the Cities Changing Diabetes (CCD) programme for Greater Manchester in partnership with and supported by Health Innovation Manchester and Novo Nordisk. CCD was launched in 2014 by the Steno Diabetes Center Copenhagen, University College London, and Novo Nordisk.

Disclaimer
The views expressed in this publication are those of the author(s) and not necessarily those of the National Institute for Health and Care Research or the Department of Health and Social Care.

Competing interests
None declared.

References

1. Galicia-Garcia, U., Benito-Vicente, A., Jebari, S., et al. Pathophysiology of Type 2 Diabetes Mellitus, *Int. Journal of Molecular Sciences*, 2020, Vol. 21:6275, doi:10.3390/ijms21176275
2. Chatterjee, S., Khunti, K. and Davies, M.J. Type 2 diabetes, *The Lancet*, 2017, Vol. 389, Issue 10085: P2239-2251, https://www.thelancet.com/journals/lancet/article/PIIS0140-6736(17)30058-2/fulltext#%20 (accessed 10/06/222)
3. Cities Changing Diabetes. Urban Diabetes: understanding the challenges and opportunities, 2016, available at: https://www.citieschangingdiabetes.com/content/dam/nnsites/ccd/en/knowledge-hub/reports/pdfs/CCD_BB_FULL_BOOK_FOR_CN_DTP.pdf (accessed 13/06/2022)
4. Mason, T., Whittaker, W., Dumville, J., et al. Estimating the ‘Rule of Halves’ for Greater Manchester: Initial analysis of data, NIHR ARC GM, University of Manchester, 2020: Manchester
5. Health Innovation Manchester. Cities Changing Diabetes: Mapping the challenge of type 2 diabetes in Greater Manchester, 2022, available at:

For peer review only - http://bmjopen.bmj.com/site/about/guidelines.xhtml

https://healthinnovationmanchester.com/wp-content/uploads/2022/06/Cities-Changing-Diabetes-Manchester-report-final-June-2022.pdf (accessed 17/06/2022)
6. Volkmann, A.M., Hesseldal, L., Napier, D. et al. Urban Diabetes Priority Assessment, 2018, available at: https://www.citieschangingdiabetes.com/content/dam/nnsites/ccd/en/knowledge-hub/urban-diabetes-toolbox/pdfs/URBAN%20DIABETES%20PRIORITY%20ASSESSMENT_Intro%20Manual_Q22019_Screen.pdf (accessed 10/06/2022)
7. Browne, J. L., Nefs, G., Pouwer, F. et al. Depression, anxiety and self-care behaviours of young adults with Type 2 diabetes: results from the International Diabetes Management and Impact for Long-term Empowerment and Success (MILES) Study, *Diabetic Medicine*, 2014, Vol. 32:133–140, DOI: 10.1111/dme.12566
8. Kunasegaran, S., Beig, J., Khanolkar, M. et al. Adherence to medication, glycaemic control and hospital attendance in young adults with type 2 diabetes, *Internal Medicine Journal*, 2018, 48: 728–731 doi:10.1111/imj.13808
9. Nanayakkara, N., Pease, A.J., Ranasinha, S., et al. Younger people with Type 2 diabetes have poorer self-care practices compared with older people: results from the Australian National Diabetes Audit, *Diabetic Medicine*, 2018, Vol. 35 (8):1087-1095, DOI: 10.1111/dme.13660
10. Nanayakkara, N., Curtis, A.J., Heritier, S., et al. Impact of age at type 2 diabetes mellitus diagnosis on mortality and vascular complications: systematic review and meta-analyses, *Diabetologia*, 2020, 64 (2):275-287. doi: 10.1007/s00125-020-05319-w.
11. Tan, N.C., Barbier, S., Lim, W.Y. et al. 5-Year longitudinal study of determinants of glycemic control for multi-ethnic Asian patients with type 2 diabetes mellitus managed in primary care, *Diabetes research and clinical practice*, 2015, Vol. 110: 218-223
12. Cities Changing Diabetes. Urban Diabetes Priority Assessment, 2018, available at: https://www.citieschangingdiabetes.com/content/dam/nnsites/ccd/en/knowledge-hub/urban-diabetes-toolbox/pdfs/URBAN%20DIABETES%20PRIORITY%20ASSESSMENT_Intro%20Manual_Q22019_Screen.pdf (accessed 30/01/2022)
13. Napier, A.D., Nolan, J.J., Bagger, M., et al. Study protocol for the Cities Changing Diabetes programme: a global mixed-methods approach, *BMJ Open*, 2017, 7:e015240. doi: 10.1136/bmjopen-2016-015240
14. Cities Changing Diabetes. Bending The Curve On Urban Diabetes: New research approaches and innovative interventions for tackling diabetes in your city, 2017, available at: https://www.citieschangingdiabetes.com/content/dam/nnsites/ccd/en/knowledge-hub/reports/pdfs/14845_CCD17_BriefingBook_A4_09102017_LR.pdf (accessed 16/06/2022)
15. Cities Changing Diabetes. Urban Diabetes Toolbox, 2022, available at: https://www.citieschangingdiabetes.com/action-framework/urban-diabetes-toolbox.html (accessed 03/08/2022)
16. Pruneddu, A. and Zentner, M. QSortware, 2012, available at: www.qsortware.com.
17. Zabala, A. Qmethod: A package to explore human perspectives using Q methodology, *R Journal*, 2014, 6 (2), 163-173. https://doi.org/10.32614/rj-2014-032 available at: https://journal.r-project.org/archive/2014/RJ-2014-032/RJ-2014-032.pdf (accessed 05/08/2022)
18. Taylor, B., Henshall, C., Kenyon, S., et al. Can rapid approaches to qualitative analysis deliver timely, valid findings to clinical leaders? A mixed methods study comparing rapid and

For peer review only - http://bmjopen.bmj.com/site/about/guidelines.xhtml

thematic analysis, *BMJ Open*, 2018, Vol. 8(10): e019993. doi:10.1136/bmjopen-2017-019993.

19. Vindrola-Padros, C., Chisnall, G., Cooper, S., et al. Carrying Out Rapid Qualitative Research During a Pandemic: Emerging Lessons From COVID-19, *Qualitative Health Research,* 2020, Vol. 30(14): 219-2204. doi.org/10.1177/1049732320951526.
20. ONS. Average household income, UK: financial year ending 2022, available at: https://www.ons.gov.uk/peoplepopulationandcommunity/personalandhouseholdfinances/incomeandwealth/bulletins/householddisposableincomeandinequality/financialyearending2022 (accessed 17/02/2023)
21. Watts, S. and Stenner, P. Doing Q Methodological Research: Theory, Method and Interpretation, London, 2012: Sage Publications Ltd
22. Walker, R.J., Smalls, B.L. and Egede, L.E. Social determinants of health in adults with type 2 diabetes—Contribution of mutable and immutable factors, *Diabetes research and clinical practice*, 2015, Vol. 110: 193-201

[Appendix 1 see separate file]

[Appendix 2 see separate file]

[Appendix 3 see separate file]

For peer review only - http://bmjopen.bmj.com/site/about/guidelines.xhtml

CCD/SC/Figure 1

“Understanding the health impact of social and cultural influences on people aged 18-40 living with type 2 diabetes: a mixed-methods study”

Figure 1. Example of a forced distribution grid (Watts & Stenner, 2012 p.17)

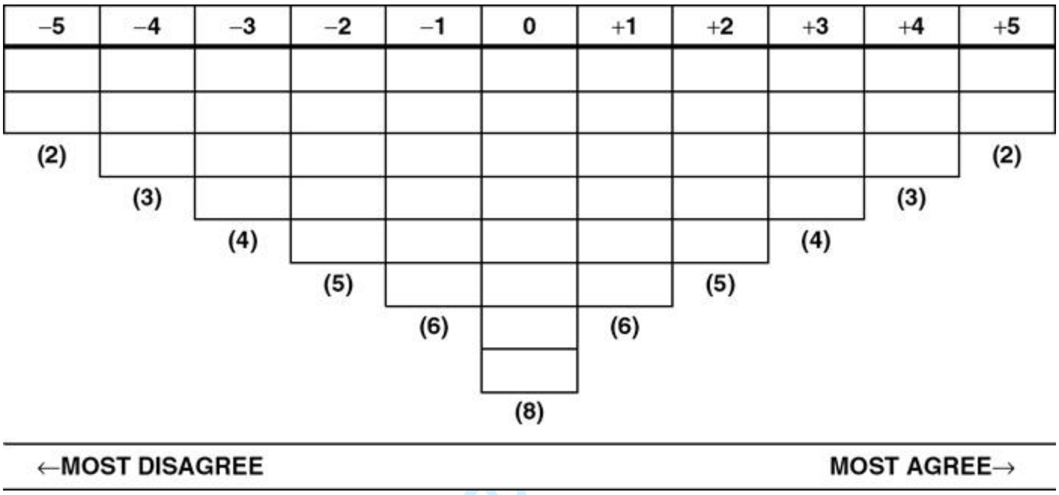

For peer review only

Appendix 1. Q-sort statements Greater Manchester factor array

	Factor 1	Factor 2	Factor 3	Factor 4	Factor 5
1 Diabetes is the least of my worries	-4	-4	0	-6	2
2 I worry about the cost of my diabetes	-3	3	-4	0	0
3 The way I make decisions about my healthcare is mostly dependent on how much money I have	-1	4	-2	3	-3
4 When it comes to food, I usually sacrifice quality for quantity	-1	-2	-4	5	6
5 Healthy foods are a luxury	-1	3	-1	1	1
6 Spending money on exercise classes or the gym is something I find difficult to do	1	5	-2	5	-1
7 Not having enough time makes people sick	1	2	-1	-4	-1
8 If I had more time, I'd exercise regularly	0	1	0	0	4
9 I just don't have the time to buy and cook healthy food	-4	1	-4	0	0
10 Diabetes support groups may be helpful, but I don't have time to participate	1	-1	2	6	-1
11 Taking care of others takes up most of my free time	0	5	-2	-2	2
12 I just don't have the skills to cook healthy meals	-3	-2	-5	1	3
13 For me, it's easy to learn about diabetes	4	0	3	-1	4
14 I think that diabetes or health-related support groups are pointless	-4	-3	0	-5	0
15 Where I live, fresh groceries are hard to get	-5	-3	-4	0	5
16 Living in the city is stressful and harmful to my health and wellbeing	-4	-1	1	-1	-5
17 I'd rather live elsewhere, but I have to live where I do because of work	-3	-4	4	2	-4
18 I am quite concerned about air pollution	0	0	0	3	-3
19 I think that where I live, it's not very safe to be out alone	-1	3	-5	-3	3
20 The climate where I live makes it really hard for me to want to walk or go somewhere by bicycle	-1	-1	-3	-1	-5
21 For me, it is important that I can get around by bike or on foot easily	0	0	-1	1	0
22 I like certain foods or dishes especially because they remind me of my childhood	1	1	1	4	-3
23 Cooking for those I love is a way of showing that I care for them	0	0	5	3	-1
24 When my family or my friends and I meet, we eat!	3	1	2	-1	-3
25 When I am by myself, I often eat food that is not very healthy	3	6	1	1	1
26 When I eat out together with friends, I usually end up eating unhealthy foods	6	4	3	-4	3
27 I often feel lonely	-5	2	2	6	2
28 To me, food is about more than just calorie intake	2	4	6	-1	3
29 Society puts a lot of pressure on women to be slim	5	5	-2	4	-3
30 I need my friends and social life to feel happy	4	0	-1	-2	-4
31 Being alone does not mean being lonely	4	2	3	0	-2
32 I tend to prioritise the needs of others over my own	3	6	0	1	-4
33 Men tend to be less interested in taking care of themselves than women	-2	-1	2	1	1
34 I have some health concerns that are more important to me than my diabetes	-2	0	1	-3	2
35 With today's medicine, diabetes is not a scary illness anymore	2	-2	3	-6	0
36 Diabetes is a disease of overweight people	-2	-5	2	-2	0
37 It is my fault that I now have diabetes	1	1	6	0	5
38 If I had known what having diabetes is like I would have tried harder to avoid becoming diabetic	2	3	0	3	-1
39 If I really had to, I could adjust to a healthy lifestyle	5	2	4	4	-2
40 I usually do what my doctor tells me to do	6	0	-1	-5	4
41 I avoid socialising because of my diabetes	-6	-3	-6	2	-1
42 I find it helpful to talk to other people about my diabetes	3	-2	-3	4	1
43 The modern world makes us ill	-1	1	3	1	-2
44 Feeling integrated with a community is important to me	1	-3	-1	3	1
45 Changes in my neighbourhood worry me	0	-1	0	0	4
46 I don't really trust health services or doctors	-6	-2	0	-5	0
47 Everybody just seems to be a bit bigger nowadays	1	0	5	0	2
48 My weight does not matter to me because I feel like a healthy person	-2	-6	-2	-3	5
49 Overweight people are often lazy	-2	-3	5	2	0
50 I am too embarrassed to go to the gym because of how I look	-1	2	0	-1	-5
51 Compared to others, I take good care of myself	0	-4	-3	-4	-2
52 I know who I can trust with my healthcare	2	0	-1	-2	1
53 I generally trust the government	0	-5	-6	-3	6
54 'Fitness freaks' have no joy in life	-3	-4	-3	2	2
55 I think that diabetes is inherited	2	1	1	-1	0
56 I think that diabetes is a death sentence	-5	-2	-2	2	-6
57 Most days, I feel good about my future	3	-1	-3	-2	3
58 At this point in my life, I feel like the choices I make are my own	5	4	4	-3	1
59 I get annoyed or upset with myself if I don't do enough for my health	2	3	1	5	-2
60 If I decide to be overweight or not exercise enough, that should be my choice	0	2	2	0	0
61 We owe it to society to be fit and healthy	0	0	1	-3	-4
62 Being unhealthy means you've given up on life	-3	-5	0	2	-1
63 Managing diabetes well is simply a matter of making the right choices	4	-1	4	-2	-2
64 My church is my rock	-2	-6	-5	0	-6
**NB: Distinguishing units are coloured**					

For peer review only

Appendix 2. Interviews and focus groups topic guide

Session	Areas to Be Covered	Example Questions/ Prompts (Not to be considered in any way exhaustive. Should be reformatted for ease of use in Focus Group or Interview session and language adapted if needed)
Introductory Session		
Open discussion around Q-Sort, content of statements, insights gained through procedure	Content of statements and nature of Q-sort	Principally unstructured discussion to give them an opportunity to voice what matters to them (and to allow participants to familiarise themselves with others in the Focus Groups).<br><br>If needed, specific sample statements may be presented as well as any qualitative comments already made. If needed, questions or prompts might include:<br>• How did you feel about X statement – is it easy to agree with/ disagree with? Why?<br>• Were there any statements that really spoke to you/ that really made you annoyed or sad? Why?<br>• Were there any statements that you did not understand or that you thought were just a bit silly? Why?<br>• Can you think of any statements that you thought were missing?<br>• The statements were based on interviews that we conducted with people in [CCD cities]. In what way do you think might their experience be similar or different to yours? Example?<br>• Were there any statements that made you reflect on how you yourself /your living with diabetes? Which ones?<br>• Did the sorting process make you think/ feel differently about yourself and your diabetes – if yes, why?<br>• Are there things we could have done differently with the Q-sort?
Discussion: Q-Sort findings		
Semi-structured discussion of findings with participants		This session provides an opportunity to deepen findings and to gather data for factor description and interpretation, as well as to identify any areas of relevance not covered by the statements. It should also be used to explore qualitatively areas of specific interest or priority. Finally, it is generally good practice to allow participants to comment on findings before finalising results.
	'Validity' of factors and exemplars	• How do you feel about [insert *defining* statements of groups]? Why?<br>• When you think about the groups (factors) that we have identified, do they seem typical/ plausible/ relevant/ well described? Why?<br>• How would you describe the various viewpoints associated with each group? Can you describe a person when you hear about these viewpoints? What would that person be like?

For peer review only

Appendix 2. Interviews and focus groups topic guide

		• What do you think of the names that we have given the groups?<br>• What do you think of the exemplars that we have described – do our descriptions make sense, do you think that this is informative/ accurate?<br>• Based on what we have presented to you, what would you say most characterizes/ differentiates the groups regarding their experience of diabetes? How does that relate to your own experiences?
	Relevance	• In what way do you think do the groups that we identified have different priorities/ abilities/ needs regarding health, wellbeing, and living with diabetes?<br>• In what way might our research findings (the groups and exemplars, description, interpretation) be useful when trying to improve diabetes care/ prevention?
	Barriers/ promoters	• How would you say do the different groups face different challenges regarding living with diabetes? What are those challenges; do you share them?<br>• What could be improved in terms of reducing [main stated barriers]?
	Social factors cultural determinants	Additional suggestion: provide list and description of social factors and cultural determinants and encourage discussion around which apply to what group; what their relevance and impact may be on each group, and how to ameliorate potentially negative impact.
Discussion: Diabetes in My City<br>Semi-structured discussion around lived experiences of participants in Greater Manchester as relevant to health, wellbeing, and diabetes		• Would you say that Greater Manchester is a good place to live for someone with diabetes? Why?<br>• Our physical activity level is important when it comes to living with diabetes. In what way does Greater Manchester make it easy or hard to be physically active? DO you have suggestions on how to change that?<br>• (If applicable: we have already discussed some things that may make living with diabetes in Greater Manchester difficult. Can you provide some more examples? How may we concretely address such issues?)<br>• If you could tell someone from [the city government/ local healthcare provider/ insurance (i.e. any potential stakeholders who may attend later session)] about something concrete they could do to improve your experience of living with diabetes, what would it be?

For peer review only - http://bmjopen.bmj.com/site/about/guidelines.xhtml

Appendix 3. Example RAP sheet

**Factor 1**	
**FGD/I participant(s):**<br>ID#<br>ID#	
**Q-sort comment(s):**<br>ID#<br>ID#<br>ID#<br>ID#	
**Analysis by:**	
**Diabetes journey**	
**Management**	
**GM lived experience**	
**Recommendations**	
**Notes (e.g. this interview a good source about x issue/standout quotations)**<br><br>ID#: “quote from transcript”<br><br>ID#: “quote from transcript”	

For peer review only

Consolidated criteria for reporting qualitative studies (COREQ): 32-item checklist

Please indicate in which section each item has been reported in your manuscript. If you do not feel an item applies to your manuscript, please enter N/A.

For further information about the COREQ guidelines, please see Tong *et al.*, 2017: https://doi.org/10.1093/intqhc/mzm042

No.	Item	Description	Section #
**Domain 1: Research team and reflexivity**			
Personal characteristics			
1.	Interviewer/facilitator	Which author/s conducted the interview or focus group?	p.6
2.	Credentials	What were the researcher's credentials? *E.g. PhD, MD*	p.1
3.	Occupation	What was their occupation at the time of the study?	p.1
4.	Gender	Was the researcher male or female?	N/A
5.	Experience and training	What experience or training did the researcher have?	p.6
Relationship with participants			
6.	Relationship established	Was a relationship established prior to study commencement?	p.5
7.	Participant knowledge of the interviewer	What did the participants know about the researcher? *E.g. Personal goals, reasons for doing the research*	N/A
8.	Interviewer characteristics	What characteristics were reported about the interviewer/facilitator? *E.g. Bias, assumptions, reasons and interests in the research topic*	N/A
**Domain 2: Study design**			
Theoretical framework			
9.	Methodological orientation and theory	What methodological orientation was stated to underpin the study? *E.g. grounded theory, discourse analysis, ethnography, phenomenology, content analysis*	p.5
Participant selection			
10.	Sampling	How were participants selected? *E.g. purposive, convenience, consecutive, snowball*	p.5
11.	Method of approach	How were participants approached? *E.g. face-to-face, telephone, mail, email*	p.5
12.	Sample size	How many participants were in the study?	p.7
13.	Non-participation	How many people refused to participate or dropped out? What were the reasons for this?	N/A
Setting			
14.	Setting of data collection	Where was the data collected? *E.g. home, clinic, workplace*	p.5-6
15.	Presence of non-participants	Was anyone else present besides the participants and researchers?	N/A

16.	Description of sample	What are the important characteristics of the sample? *E.g. demographic data, date*	p.5
Data collection			
17.	Interview guide	Were questions, prompts, guides provided by the authors? Was it pilot tested?	p.6
18.	Repeat interviews	Were repeat interviews carried out? If yes, how many?	N/A
19.	Audio/visual recording	Did the research use audio or visual recording to collect the data?	p.6
20.	Field notes	Were field notes made during and/or after the interview or focus group?	N/A
21.	Duration	What was the duration of the interviews or focus group?	p.6
22.	Data saturation	Was data saturation discussed?	N/A
23.	Transcripts returned	Were transcripts returned to participants for comment and/or correction?	N/A
**Domain 3: analysis and findings**			
Data analysis			
24.	Number of data coders	How many data coders coded the data?	p.13
25.	Description of the coding tree	Did authors provide a description of the coding tree?	N/A
26.	Derivation of themes	Were themes identified in advance or derived from the data?	p.7
27.	Software	What software, if applicable, was used to manage the data?	p.6
28.	Participant checking	Did participants provide feedback on the findings?	N/A
Reporting			
29.	Quotations presented	Were participant quotations presented to illustrate the themes / findings? Was each quotation identified? *E.g. Participant number*	p.7-9
30.	Data and findings consistent	Was there consistency between the data presented and the findings?	p.7-10
31.	Clarity of major themes	Were major themes clearly presented in the findings?	p.9-10
32.	Clarity of minor themes	Is there a description of diverse cases or discussion of minor themes?	p.11-12

When submitting your manuscript via the online submission form, please upload the completed checklist as a Figure/supplementary file.

If you would like this checklist to be included alongside your article, we ask that you upload the completed checklist to an online repository and include the guideline type, name of the repository, DOI and license in the *Data availability* section of your manuscript.

Developed from: Allison Tong, Peter Sainsbury, Jonathan Craig, Consolidated criteria for reporting qualitative research (COREQ): a 32-item checklist for interviews and focus groups, International Journal for Quality in Health Care, Volume 19, Issue 6, December 2007, Pages 349–357, https://doi.org/10.1093/intqhc/mzm042

For peer review only

BMJ Open

What are the perspectives of adults aged 18-40 living with type 2 diabetes in urban settings towards barriers and opportunities for better health and wellbeing: a mixed-methods study

Journal:	*BMJ Open*
Manuscript ID	bmjopen-2022-068765.R2
Article Type:	Original research
Date Submitted by the Author:	19-Aug-2023
Complete List of Authors:	Croke, Sarah; The University of Manchester,<br>Volkmann, Anna-Maria; University College London, Anthropology<br>Perry, Catherine ; The University of Manchester, Division of Population Health, Health Services Research and Primary Care<br>Atkinson, Ross; University of Manchester, Manchester, M13 9PL , Division of Nursing, Midwifery and Social Work, School of Health Sciences, Faculty of Biology, Medicine and Health;<br>Pruneddu, Alessio; Independent research consultant<br>Morris, Lydia; The University of Manchester, Division of Psychology and Mental Health, School of Health Sciences<br>Bower, Peter; University of Manchester, NPCRDC
**Primary Subject Heading**:	Diabetes and endocrinology
Secondary Subject Heading:	Health services research
Keywords:	PRIMARY CARE, QUALITATIVE RESEARCH, DIABETES & ENDOCRINOLOGY

SCHOLARONE™
Manuscripts

I, the Submitting Author has the right to grant and does grant on behalf of all authors of the Work (as defined in the below author licence), an exclusive licence and/or a non-exclusive licence for contributions from authors who are: i) UK Crown employees; ii) where BMJ has agreed a CC-BY licence shall apply, and/or iii) in accordance with the terms applicable for US Federal Government officers or employees acting as part of their official duties; on a worldwide, perpetual, irrevocable, royalty-free basis to BMJ Publishing Group Ltd ("BMJ") its licensees and where the relevant Journal is co-owned by BMJ to the co-owners of the Journal, to publish the Work in this journal and any other BMJ products and to exploit all rights, as set out in our licence.

The Submitting Author accepts and understands that any supply made under these terms is made by BMJ to the Submitting Author unless you are acting as an employee on behalf of your employer or a postgraduate student of an affiliated institution which is paying any applicable article publishing charge ("APC") for Open Access articles. Where the Submitting Author wishes to make the Work available on an Open Access basis (and intends to pay the relevant APC), the terms of reuse of such Open Access shall be governed by a Creative Commons licence – details of these licences and which Creative Commons licence will apply to this Work are set out in our licence referred to above.

Other than as permitted in any relevant BMJ Author's Self Archiving Policies, I confirm this Work has not been accepted for publication elsewhere, is not being considered for publication elsewhere and does not duplicate material already published. I confirm all authors consent to publication of this Work and authorise the granting of this licence.

For peer review only

- Title of the article

"What are the perspectives of adults aged 18-40 living with type 2 diabetes in urban settings towards barriers and opportunities for better health and wellbeing: a mixed-methods study"

- Full name, postal address and e-mail of the corresponding author

1. Dr Sarah Croke, PhD

Centre for Primary Care and Health Services Research, Division of Population Health, Health Services Research & Primary Care, School of Health Sciences, Faculty of Biology, Medicine and Health, University of Manchester, Suite 1 6th Floor Williamson Building, Oxford Road, Manchester, UK, M13 9PL. tel: 07932 011581 email: sarah.croke@manchester.ac.uk

- Full name, department, institution, city and country of all co-authors

2. Dr Anna-Maria Volkmann, PhD

Department of Anthropology, University College London, London, UK. email: a.volkmann@ucl.ac.uk

3. Dr Catherine Perry, PhD

NIHR Applied Research Collaboration Greater Manchester (NIHR ARC GM), Division of Population Health, Health Services Research & Primary Care/Division of Nursing, Midwifery & Social Work, School of Health Sciences, Faculty of Biology, Medicine and Health, University of Manchester, Manchester, UK. email: catherine.perry@manchester.ac.uk

4. Dr Ross A. Atkinson, PhD

NIHR Applied Research Collaboration Greater Manchester (NIHR ARC GM), Division of Nursing, Midwifery & Social Work, School of Health Sciences, Faculty of Biology, Medicine and Health, University of Manchester, Manchester, UK. email: ross.atkinson@manchester.ac.uk

5. Dr Alessio Pruneddu, PhD

Independent research consultant, Salford, UK. email: info@qsortouch.com

6. Dr Lydia Morris, PhD

Division of Psychology and Mental Health, School of Health Sciences, Faculty of Biology, Medicine and Health, University of Manchester, Manchester, UK. email: lydia.morris@manchester.ac.uk

7. Professor Peter Bower, PhD

NIHR Applied Research Collaboration Greater Manchester (NIHR ARC GM), Centre for Primary Care and Health Services Research, Division of Population Health, Health Services Research & Primary Care, School of Health Sciences, Faculty of Biology, Medicine and Health, University of Manchester, Manchester, UK. email: peter.bower@manchester.ac.uk

- Word count, excluding title page, abstract, references, figures and tables

4777 (excludes participant quotes as results).

For peer review only - http://bmjopen.bmj.com/site/about/guidelines.xhtml

For peer review only

"What are the perspectives of adults aged 18-40 living with type 2 diabetes in urban settings towards barriers and opportunities for better health and wellbeing: a mixed-methods study"

Sarah Croke, Anna-Maria Volkmann, Catherine Perry, Ross A. Atkinson, Alessio Pruneddu, Lydia Morris, and Peter Bower.

Abstract

Objectives: Delivered as part of the global assessment of diabetes in urban settings, this study explores different aspects of living with type 2 diabetes, for adults aged 18-40. Primary questions were: 1) can we identify sub-groups of adults under 40 years old sharing specific perspectives towards health, wellbeing and living with type 2 diabetes; and 2) do these perspectives reveal specific barriers to and opportunities for better type 2 diabetes prevention and management and improved wellbeing?

Design*:* The study employed a mixed method design with data collected through demographic questionnaires, Q-sort statement sorting exercises, focus groups discussions and individual interviews.

Setting: Primary care across Greater Manchester, UK.

Participants*:* Those aged between 18 and 40, with a confirmed type 2 diabetes diagnosis, and living in Greater Manchester were eligible to participate. A total of 46 people completed the Q-sort exercise and 43 were included in the final analysis. Of those, 29 (67%) identified as female and 32 (75%) as white. Most common time since diagnosis was between five and ten years.

Results: The Q-sort analysis categorised 35 of the 43 participants (81%) into five sub-groups. Based on average statement sorts for each sub-group, perspectives were characterised as: 1) Stressed and calamity coping (n=13), 2) Financially disadvantaged and poorly supported (n=12), 3) Well-intentioned but not succeeding (n=5), 4) Withdrawn and worried (n=2), 5) Young and stigmatised (n=3). Holistic analysis of our qualitative data also identified some common issues across these sub-groups.

Conclusions: Adults under 40 with type 2 diabetes are not a homogenous group, but fall into five identifiable sub-groups. They also experience issues specific to this age group that make it particularly difficult for them to focus on their own health. More tailored support could help them to make the necessary lifestyle changes and manage their type 2 diabetes better.

For peer review only - http://bmjopen.bmj.com/site/about/guidelines.xhtml

Article Summary

Strengths and limitations of this study

- A key feature of a Q-study is the ability to reveal shared perspectives among a group of participants, and for those to be understood holistically.
- The Q-sort requires a minimum of 35 participants to ensure statistical validity and a maximum of 64 (i.e. not exceeding the number of statements).
- The challenge to recruit younger adults living with type 2 diabetes as participants for this study were addressed by utilising multiple routes, including social media.
- Restrictions due to the COVID-19 pandemic forced us to conduct all data collection via online platforms; this limited some from participating while providing opportunity to others.
- Zabala's 'qmethod' analysis package allowed us to extract factors meeting statistical significance (high loading score on a single factor with $p<0.05$), and thereby establish shared perspectives in our sample despite sometimes having smaller numbers within some sub-groups.

For peer review only

For peer review only - http://bmjopen.bmj.com/site/about/guidelines.xhtml

Introduction

Type 2 diabetes is one of the most common metabolic disorders worldwide [1] and leads to complications that cause both psychological and physical distress and put a huge burden on healthcare systems [2]. One response to this, in recent years, has been the Cities Changing Diabetes programme, a programme advocating for urgent action against type 2 diabetes on a global scale. Launched in 2015, via a partnership between Novo Nordisk, University College London and Steno Diabetes Center, and implemented by City Partners, it aims to map the challenge, share solutions and act on the growing impact of type 2 diabetes in some of the world's great cities [3]. In 2019, Greater Manchester, UK became a City Partner.

A report conducted as part of this programme using the 'Rule of Halves' approach [4, 5] identified patterns of diagnosis and treatment in Greater Manchester and indicated that type 2 diabetes in the area is under-diagnosed in adults under 40 years of age. This also showed that, where type 2 diabetes was diagnosed, those under 40 seem less likely to receive appropriate care and achieve HbA1c treatment targets when compared with older age groups. Building on this, we conducted the Urban Diabetes Priority Assessment to explore shared priorities, attitudes, and points of view among local people with type 2 diabetes. Shared perspectives are important for informing future interventions and policies, while also helping to strengthen the global Cities Changing Diabetes research platform for understanding the social-cultural drivers of Diabetes [6].

Previous research suggests that adults aged 18-40 with type 2 diabetes have higher physical morbidity and mortality than other type 2 diabetes sub-groups and poorer outcomes than their older counterparts [7]. People in this group are also more likely to be obese with sub-optimal glycaemic control and have a high risk of developing at least one severe diabetes complication by the time they are in their 40s, reducing life expectancy by 15 years [7]. A further concern is poor adherence to medication [8, 9]. Being a younger age at diagnosis is also associated with higher risk of mortality and vascular disease [10], while people may require more intensive psychological and self-care support compared with those who develop type 2 diabetes later [7]. It is suggested that targeting younger patients, earlier in their trajectory, can present a unique opportunity to improve longer term outcomes [11].

We aimed to explore the following questions for adults under 40 with type 2 diabetes, living in Greater Manchester:

- Can we identify sub-groups of adults under 40 years old sharing specific perspectives towards health, wellbeing and living with type 2 diabetes?
- Do these perspectives reveal specific barriers to and opportunities for better type 2 diabetes prevention and management and improved wellbeing?

For peer review only - http://bmjopen.bmj.com/site/about/guidelines.xhtml

Methods

Study design

The present study is part of the Cities Changing Diabetes protocol [12], which forms part of a wider global mixed methods approach [13]. This study draws on the principles of Q-methodology, a technique increasingly used by researchers to explore health-related decision-making and behaviours via the systematic and scientific study of subjective viewpoints. The aim of a Q-study is to extract as many world-views as possible, obtaining perspectives and point of views from highly informed individuals rather than from a sample representative of the general population, and employs a mixture of data collection and analyses to obtain insights [14]. It follows the earlier 'Rule of Halves' analysis and delivers the 'Urban Diabetes Priority Assessment' [15] which consists of two components.

The first component required participants to complete an online demographic questionnaire and computer based Q-sort, where participants prioritise a set of 64 statements according to their personal preference. The statements in the Q-sort reflect eight social factors and cultural determinants of diabetes vulnerability and were generated from prior Cities Changing Diabetes Vulnerability Assessment studies conducted in five global cities: Copenhagen, Mexico City, Houston, Shanghai and Tianjin. Across those sites, 746 individual assessments were carried out by AMV and University College London (UCL) colleagues. Each assessment lasted on average two hours and centred around a specific interview guide which had been translated and locally-adapted. The assessments also captured demographic data via a survey as well as ethnographic observation data [13]. Examples of statements include: "Healthy foods are a luxury", "For me, it's easy to learn about diabetes", "I tend to prioritise the needs of others over my own", and "I think that diabetes is a death sentence" (full list in appendix 1). The local research teams as well as the global team (UCL) shared consensus that they adequately reflected the lived experience of the participants in the Cities Changing Diabetes Vulnerability Assessment. The statement set was first validated as part of the Cities Changing Diabetes Vancouver Q-Sort study, and further validated as part of the Cities Changing Diabetes Moscow Q-sort Study.

The second component employed focus group discussions and individual interviews to facilitate in-depth exploration of the outcome of the Q-sort with selected participants [6].

Participants and recruitment

Adults aged between 18 and 40, with a confirmed type 2 diabetes diagnosis, and living in Greater Manchester were eligible to participate. The decision to focus recruitment on the 18-40 age group reflected findings from the Greater Manchester 'Rule of Halves' analysis. Participants were recruited through a combination of approaches: invitation by their GP or pharmacy; invitation via a consent-to-contact database (Help BEAT Diabetes); articles in Diabetes UK newsletters; enlisting community leaders within South Asian communities; and direct advertising on social media platforms (Facebook, Instagram, Twitter). Interested participants contacted researchers whereupon they were screened using a stratified sampling approach to ensure a diverse range of experiences, in regard to age, gender, ethnicity and socioeconomic status.

Data collection

For peer review only - http://bmjopen.bmj.com/site/about/guidelines.xhtml

After giving informed consent, eligible participants were granted access to our online data collection tool (Qsortware) [16]. Initially, participants completed a demographic survey (socioeconomic status, housing, employment, health insurance, long-term conditions, diabetes complications, height and weight). Next they undertook a statement sorting exercise (‘Q-sort’) to assess their perspectives on living with type 2 diabetes. Finally, those who had consented to be contacted were invited for stage 2 interviews and focus groups. Participants who agreed joined others to explore the results from the computer-based data collection, adding depth and detail to the statistical data, following a Cities Changing Diabetes-specific interview protocol [14].

Q-sort

The 64 statements had been synthesised from participant interviews in prior studies and represent a broad range of social and cultural factors relevant to health, well-being and type 2 diabetes [13, 14]. The social factors were financial, time, resource, and geographic constraints and the cultural factors were traditions and conventions, health and illness, change and transition, self and other, and agency and choice [3].

In the sorting exercise, participants were instructed to rank statements based on their individual level of agreement or disagreement with each statement. In the first step (preliminary sort), participants were asked to sort statements into three “piles” (agree/disagree/neutral). In the second step (the final sort), participants added statements into a forced distribution grid (the Q-sort - see figure 1). We encouraged participants to add open-ended comments about each statement in a final step.

Figure 1. Example of a forced distribution grid (Watts & Stenner, 2012 p.17)

[see separate file]

Interviews & Focus Groups

The interviews and focus groups were led by an experienced qualitative health researcher (SC) with one or more other experienced researchers (AMV, CP and RAA) in support, conducted remotely via secure video conferencing software (e.g. Zoom or MS Teams), digitally recorded (audio only) and professionally transcribed verbatim. Topic guides covered the journey to diagnosis, type 2 diabetes management, and the experiences of living with type 2 diabetes in Greater Manchester (see appendix 2). We also explored areas where support was lacking and recommendations that could improve the experience of being a younger adult with type 2 diabetes. Focus Groups typically lasted 90 minutes and Interviews lasted 60 minutes.

Data Analysis

We used Zabala’s ‘qmethod’ analysis package [17], run in R, to extract factors from the Q-sort and explore shared perspectives in our sample. To be assigned to a factor, participants needed a high loading score on a single factor, with p<.05, and for this squared score to be higher than the squared sum of the scores obtained in all the other factors. Factors were described by the agreement, disagreement and neutrality expressed for each statement, both when compared to the other subgroup decision on that statement and in relation to the agreement/disagreement of the other statements of the Q-set, since there was a forced choice distribution involved. As is customary in Q-methodology, the research team (AMV, AP, SC, CP, RAA) discussed various solutions and used the ‘factor array’ (see appendix 1) to identify defining statements for each sub-group. A series of T-tests identified those statements that each subgroup sorted out differently, therefore helping the

For peer review only - http://bmjopen.bmj.com/site/about/guidelines.xhtml

interpretation. However, even those statements considered 'consensus' were still relevant, since they informed about issues/ barriers universally perceived as significant by each perspective. ‘Crib sheets’ were used to extract the characteristics of each sub-group from 1) statements that ranked the highest in the factor compared to other factors; 2) statements that ranked the lowest in the factor compared to the other factors; 3) statements that ranked at -6 (very strongly disagree) ; and 4) statements that ranked at +6 (very strongly agree). Interpretation of the factors began by taking together all the statements in the ‘strongly agree' area and those in the ‘strongly disagree' area, especially when this configuration is in stark contrast with the other sub-groups. The configuration of items and their interrelationships provided the basis for initial interpretations of participant perspectives for each sub-group.

Thematic coding in NVivo12 and rapid assessment procedure (RAP) sheets [18, 19] were used to provide further, in-depth understanding of the issues that underpin the Q-sort and to supplement initial interpretations for each sub-group (see appendix 3). These were synthesised with the demographic data and the open-ended comments from the Q-sort, to provide a comprehensive overview.

Patient and public Involvement

There was no patient involvement in the design or planning of the study. We made several attempts to recruit younger adults living with type 2 diabetes as patient and public contributors, to advise on delivery and dissemination of this study, however this was unsuccessful within the timelines. This reflected the challenges of the COVID-19 pandemic and a general lack of engagement by this age group with formal health and community support services (e.g. Diabetes UK; Help BEAT Diabetes; local community champions).

For peer review only

For peer review only - http://bmjopen.bmj.com/site/about/guidelines.xhtml

Results

From 46 completions, 43 participants provided analysable data in the Q-sort. Of these, 17 (40%) participated in a focus group or interview. Table 1 shows a summary of participant characteristics.

Table 1. Summary of participants (n = 43)

Participant characteristic		
Age (years)	Mean	35.3
	Standard Deviation	4.4
Sex	Male	67%
	Female	33%
Body Mass Index (n=41)	Healthy weight (BMI 18.5 – 24.9)	12.2%
	Overweight (BMI 25 – 29.9)	7.3%
	Obese (BMI 30 – 39.9)	41.5%
	Severely obese (BMI over 40)	39.0%
Ethnicity	White	74.4%
	Asian/Asian British	20.9%
	Black/African/Caribbean/Black British	2.3%
	Multiple Ethnic Groups	2.3%
Household income (£)	<£30k	46.5%
	≥£30k	53.5%

We extracted five factors (representing 81% of participants) to create our sub-groups. Determining the best factor solution resulted from several discussions of the many variables involved, the complexity of the topic, and after excluding every factor solution with factors based on a single individual. We considered the total variance explained, the correlation between factors, the eigenvalues, and reliability coefficients for each factor (Humphrey's rule). Individual Q-sorts were assigned to a factor according to specific criteria set by 'q-method', the library from R we used for data analysis. This activity, called 'flagging', ensured each factor was highly distinguishable from the other ones extracted. All assigned participants achieved a high loading score on a single factor with $p<0.05$ and with the squared score higher than the squared sum of the scores obtained in all the other factors. We opted for a five factor solution since the variance explained was maximised in a stable statistical structure.

Based upon their characteristics, we named these sub-groups 1) Stressed and calamity coping (n=13), 2) Financially disadvantaged and poorly supported (n=12), 3) Well-intentioned but not succeeding (n=5), 4) Withdrawn and worried (n=2), 5) Young and stigmatised (n=3). These interpretations reflect the key viewpoints captured by each study factor created from their relative and distinguishing statement rankings and demographic information, illustrated by the interview and focus group data [20]. They are woven and presented in a *narrative* style to present a seamless account of each sub-group's holistic viewpoint.

Holistic analysis of our qualitative data also identified some common issues across these five identifiable sub-groups: overwhelming care obligations in those with young families; early or mid-career work pressures and stress; exclusion from social life; experiences with health professionals; and lack of specific skills and knowledge that could support them better. Some of these may be more relevant to adults aged 18-40 than other age groups and suggest a gap within current health and support services. Ethnicity had no clear influence on participant fit to sub-groups. Household

For peer review only - http://bmjopen.bmj.com/site/about/guidelines.xhtml

For peer review only

income showed potential influence in two of the sub-groups: in sub-group 3 almost all participants reported incomes over £30k, while in sub-group 4 all participants reported incomes under £30k, a figure which is broadly in line with the current UK median household income [21]. Eight participants did not appear to fit any sub-group. These individuals still shared some common experiences with those in the five sub-groups, including observations regarding ethnicity and household income, and two participated in qualitative interviews. Their inclusion in our holistic analysis provides additional interpretation and contextualisation.

Sub-group 1: 'Stressed and calamity coping' (n=13; qualitative interviews n=7)

These participants were mostly female (average age 34). Ten identified as white and three identified as Asian/Asian British. Their key viewpoint is that friends and family are very important. Despite initial challenges at diagnosis, they report having good awareness and some ability to manage their diabetes. They often ignore the onset of type 2 diabetes, and can be unclear about early symptoms.

> "I don't know how long I was diabetic for, I wouldn't be surprised if I've been diabetic for at least a couple of years before my diagnosis. Now it's one of those cases where you realise signs were there but they weren't as prominent" (a male ages 20-30 years old, BMI 51)

They expressed a genuine desire to 'turn things around' but managing other demands (including stress, work and dependants) often made it difficult. Self-described 'food addiction' was also a feature.

> "Yeah, because I think food addiction is very real but there is no recognition for it really anywhere." (a female ages 30-40 years old, BMI 43)

Sub-group 2: 'Financially disadvantaged and poorly supported' (n=12; qualitative interviews n=4)

Participants in this sub-group were mostly female (average age 36). Ten identified as white, one identified as Asian/Asian British and one identified as Black/African/Caribbean/Black British. Their key viewpoint is they prioritise the needs of others, at the expense of themselves. They report lacking skills, finances and support to better manage their type 2 diabetes and may already have some complications. People are often 'pre-diabetic' for some time before being diagnosed with type 2 diabetes. Following diagnosis, they can feel unsure about how to manage their symptoms and do not always know how to find further information.

> "But there was nothing, it was just like right, well, no potatoes, come off the coke, do more exercise, see you later". (a female ages 30-40 years old, BMI 50)

They would like more support with diet; although time, skills and money for healthy eating and exercise are barriers. They also perceive a clinical focus on medication, despite struggling with side-effects. They will often felt embarrassed, self-conscious and judged by others.

> "I haven't really told everybody either. I haven't told my partner; he doesn't know. I've told my mum but I think it's the same, it's that you feel like people are going to judge you for having it and it's your fault because of the way that you eat." (a female ages 30-40 years old, BMI 40)

Sub-group 3: 'Well-intentioned but not succeeding' (n=5; qualitative interviews n=3)

These participants were mostly male (average age 37). Three identified as white, one identified as Asian/Asian British and one identified as multiple ethnic groups. Their key viewpoint is they feel responsible for having diabetes, and so are responsible for managing it. They report being proactive in managing their health and trying to balance their diagnosis within their wider circumstances and

For peer review only - http://bmjopen.bmj.com/site/about/guidelines.xhtml

lifestyles. Some regard being overweight as 'socially acceptable' and can have already developed associated complications. Often diagnosed as 'pre-diabetic' prior to full onset, they may report a family history of diabetes and expect a diagnosis sooner or later; although they are surprised to be diagnosed at such a young age.

> "It's my turn sooner or later it would be anyway." (a male ages 30-40 years old, BMI 29)

Family responsibilities are viewed as a positive motivation for change, and they feel that diet is more important than exercise in controlling type 2 diabetes. They like to ask a lot of questions and are quite optimistic about the future.

> "I think it's in moderation, and to be quite sensible with things, and what works for you. I've given up smoking, probably about five or six years too late, and again, because of my daughter, and priorities and things like that." (a male ages 30-40 years old, BMI 29).

Sub-group 4: 'Withdrawn and worried' (n=2; qualitative interviews n=0)

These participants were both male (average age 35). One identified as white and one identified as Asian/Asian British. Their key viewpoint is being isolated with concerns about future health. They find type 2 diabetes scary and say they would have tried to avoid it, if they had known more about it earlier. They are unsure whom to trust with their healthcare and/or advice. They find it hard to learn about their type 2 diabetes and do not feel well equipped to take care of themselves. They feel their choices are limited, increasing anxiety about their health. They find it hard to socialise (because of diabetes) which increases feelings of loneliness.

In the absence of interview and focus group data, this interpretation and identification of key viewpoint relies solely on their relative and distinguishing statement rankings, plus demographic information.

Sub-group 5: 'Young and stigmatised' (n=3; qualitative interviews n=1)

Participants were a mix of female and male (average age 33). Two identified as white and one identified as Asian/Asian British. Their key viewpoint is there is more to life than diabetes. They report being too young to worry too much about complications, although may have already experienced serious complications (e.g. acute kidney injury). They feel embarrassment about having type 2 diabetes so young, and find it hard to accept their diagnosis.

> "One of the doctors were like, you can go blind and you can…and I'm like yeah, I'm not going to go blind, I'm not 60 yet." (a female ages 20-30 years old, BMI 24)

They believe type 2 diabetes can be reversed, with diet and exercise, but it is hard without the right environment and support. They struggle to tolerate side-effects of medications, and choose non-adherence where they feel these are not compatible with their age and lifestyle. There is some resentment towards health services.

> "I was like I can't even socialise, I can't go even go out. And especially when I was younger I was like I'm not doing this. So, I stopped taking the injections at that point." (a female ages 20-30 years old, BMI 24)

Common issues

For peer review only
For peer review only - http://bmjopen.bmj.com/site/about/guidelines.xhtml

In addition to identifying sub-groups who share specific perspectives towards health, wellbeing and living with type 2 diabetes, we also identified common issues across the sub-groups that create barriers to better type 2 diabetes prevention, management and improved wellbeing.

Overwhelming care obligations in those with young families

We learned the busy nature of peoples' lives often meant that, whatever their different situations, making the best choices was not easy. People across all sub-groups reported many barriers to lifestyle improvements. The combination of long working hours and childcare responsibilities made many people feel so tired at the end of the day that they would often reach for convenience foods. A lack of time, cooking skills or money were also reasons for less healthy choices. We saw signs of a gender split, with males largely citing family responsibilities as their motivation to 'do better', while females largely found these a barrier. Family life events and balancing responsibility for older and younger generations were a common source of stress.

Early or mid-career work pressures and stress

Many participants told us they struggled to accept their diagnosis at such a young age. They often had other priorities, such as socialising, progressing their careers and getting on the housing 'ladder'. Long working hours made it difficult to access exercise classes or gyms at quieter and cheaper times. For some, working patterns and environments made it difficult to take regular breaks, eat healthily or ask for more support. The workplace was viewed as somewhere people feel they could be better supported. A lack of employer support made it more difficult to adopt good type 2 diabetes management.

Exclusion from social life

A number of our participants spoke about feeling embarrassed or ashamed of being diagnosed with type 2 diabetes, or admitting their diagnosis to others. They often experienced unpleasant side effects from medication (flatulence, diarrhoea etc) which could impact their social life, and being self-conscious about body size/shape could prevent access to exercise classes. Many expressed a desire to engage more with other people with type 2 diabetes, via support or exercise groups, suggesting they felt social support to be beneficial but lacking.

Experiences with health professionals

People shared with us a range of journeys to diagnosis, including family history, gestational diabetes, and pancreatic injury. Some had a period of 'pre-diabetes' prior to diagnosis. Those having a type 2 diabetes diagnosis at a younger age felt that health professionals did not always listen to them or provide appropriate services or support, suited to their needs. Medication as a primary treatment was not popular with participants, yet few alternatives were presented when common medications caused intolerable side-effects. This often led to refusal to take any prescribed medications. The perceived emphasis on services for older people with type 2 diabetes left many feeling abandoned or stigmatised. Previous negative experiences with health professionals could also influence current experiences, and contributed to feelings that younger people do not get as much support as older people. People in all sub-groups felt they had not received enough education about 'being healthy' or recognising the 'signs of type 2 diabetes' prior to onset. The absence of formal support increased use of and dependency on internet sources, self-help books and anecdotal advice, which may not always be appropriate. A desire to 'reverse' or manage their type 2 diabetes with food and exercise, in the first instance, was met with a lack of support from health professionals. Health services were regarded as good quality once people were advanced into the 'complications' phase of type 2

For peer review only - http://bmjopen.bmj.com/site/about/guidelines.xhtml

diabetes, but poor in prevention and early management. Provision was perceived as better in surrounding towns and worse in the city.

Lack of specific skills or knowledge that could support them better

We found that, despite their younger age, participants in our sample often had very high BMIs – over 80% reporting data were classed as obese (BMI over 30) or severely obese (BMI over 40). However, many reported not having the skills or knowledge to manage their health alone. For those with young children this was a cause of additional concern, as it meant their children would be left to witness and/or deal with any crisis (hypo) episodes, and they were also concerned their children may repeat their habits. A number of people we spoke to had parents or older relatives who already had type 2 diabetes. The current provision of pre-diabetes and type 2 diabetes services was not felt to provide the skills or knowledge to meet the different needs of younger people. Younger adults across the sub-groups noted how their finances and commitments and daily schedules are different to many older adults.

For peer review only

Discussion

This study aimed to identify and understand the perspectives and experiences of younger adults living with type 2 diabetes in Greater Manchester. We found five sub-groups which illustrated the everyday challenges people face and the different strengths they may have to moderate these. These sub-groups cut across socioeconomic factors such as household income, employment status, ethnicity, housing type, and residence ownership. We also found common challenges related to this age group.

Strengths and limitations

A key feature of a Q-study is the ability to reveal shared perspectives among a group of participants, and for those to be understood holistically [20]. The Q-sort requires a minimum of 35 participants to ensure statistical validity and a maximum of 64 (i.e. not exceeding the number of statements). We faced some challenges in recruiting younger adults living with type 2 diabetes as participants for this study, which thereby extended our recruitment period. This may reflect both the COVID-19 pandemic and a general lack of engagement by this age group with formal health services. To address this, we utilised multiple routes to optimise recruitment. In addition, the restrictions of the COVID-19 pandemic forced us to conduct all data collection via online platforms, which limited some from participating while providing opportunity to others. Zabala's 'qmethod' analysis package [17] allowed us to extract factors meeting statistical significance (high loading score on a single factor with $p<0.05$), and thereby establish shared perspectives in our sample, despite sometimes having smaller numbers within some sub-groups. Stratified sampling ensured we had a diverse range of experiences, with regard to age, gender, ethnicity and socioeconomic status, represented.

Comparison with the wider literature

Previous literature focusing on type 2 diabetes with a younger adult age group have revealed some concerning data on trends and issues. To our knowledge, this is the first Q-sort study in this population.

Browne et al [7] have previously noted that younger adults are more likely to be obese with sub-optimal glycaemic control and have a higher risk of developing at least one severe diabetes complication by their 40s. We found that people across all sub-groups had very high BMI and that self-reported poor glycaemic control was common. Reasons for this varied between the sub-groups, however the multiple pressures, unacceptability of side effects, and a lack of appropriate support were all felt to contribute.

Kunasegaran et al [8] and Nanayakkara et al [9] have both highlighted the issue and need to understand better the barriers patients face in regard to medication adherence, the impact of this on glycaemic control and how this can be improved. Browne et al [7] have also suggested that people aged ≤ 45 years may require more intensive psychological and self-care support compared with those who develop type 2 diabetes aged ≥ 45 years. From our study, poor toleration of medications and their side-effects was a common issue raised by people across all sub-groups. Others suggested a preference for non-pharmaceutical approaches (diet, counselling and exercise) as a primary approach but found little support from statutory services, and some simply felt they 'still had time' before needing to submit to control through medication.

Tan et al [11] and Nanayakkara et al [10] have previously called for effective targeting of younger patients, earlier in their journey, as longer type 2 diabetes duration increases risk of complications. We found that many people, including those with a family history of type 2 diabetes, did not know

For peer review only - http://bmjopen.bmj.com/site/about/guidelines.xhtml

how to recognise early symptoms, or could easily confuse these with other causes. Those with a family history also believed it was to some extent inevitable they would develop type 2 diabetes, regardless of their habits, although only expected this when they were older. People without a family history were often ashamed and considered it their fault for developing type 2 diabetes, as they had not followed healthier lifestyles earlier. The lack of appropriate education, both in schools and in pre-diabetes courses, was felt to have contributed to this situation.

Walker et al [22] used a quantitative approach to examine the contribution of social determinants on glycaemic control in all adults (aged 18+) and found interventions that target self-efficacy and social support lead to improvements in glycaemic control, while clinical interventions can lower the burden of disease and address mutable social determinants. The overall age of people in our study was younger, however, we also found that greater social support and personalisation were proposed as potential solutions to common issues.

Implications of the study

Across our sub-groups, our findings suggest a perception of a lack of personalisation of diabetes support for those aged 18-40. People living with type 2 diabetes at a younger age want to be healthier, fitter, and more engaged in their own care. They frequently worried about their health problems and were anxious about the future. A lack of targeted programmes, that take their particular circumstances into account, make it harder to prioritise their type 2 diabetes care and achieve the necessary 'lifestyle modification' to prevent, delay or manage type 2 diabetes well.

The people we spoke to shared some common experiences/problems, but they each appeared to require different solutions. Younger people with type 2 diabetes need their GPs and care providers to recognise that they are not just individual patients, but are often parents and/or partners that live in complex and busy households. In addition, while much type 2 diabetes prevention and management is currently structured towards an older population, people who develop type 2 diabetes during their teens, twenties and thirties will ultimately need to manage this condition for longer, raising their risk of serious complications in the future. The condition is also heavily stigmatised and younger patients may suffer disproportionately from this.

As our primary purpose was to determine if we could identify sub-groups of the 18-40 population who share perspectives, we did not specifically explore the impact of ethnicity or socioeconomic differences, with regards to the experience of the respondents or service access. We suggest further research could explore these issues.

Conclusion

The Rule of Halves study that informed our work, as part of the Cities Changing Diabetes protocol, suggested that in Greater Manchester younger adults are under-diagnosed, less likely to receive appropriate care and less likely to achieve their HbA1c treatment targets compared with older age groups. Using Q-methodology, we have explored the experiences of younger adults with type 2 diabetes, in Greater Manchester, identifying discrete sub-groups within this population and providing insights to different approaches that might help them avoid, delay onset or live well with type 2 diabetes. We also identify some common issues across the sub-groups that may impact their long term trajectory of type 2 diabetes and found particular patterns of lifestyle and social challenges that could inform services how to be better designed, to be sensitive to the needs and preferences of particular groups, including how these are advertised, delivered and assessed.

For peer review only - http://bmjopen.bmj.com/site/about/guidelines.xhtml

For peer review only

For peer review only - http://bmjopen.bmj.com/site/about/guidelines.xhtml

Acknowledgements
The authors would like to thank Diabetes UK and Help BEAT Diabetes, hosted by Research for the Future (RftF): see https://www.ncbi.nlm.nih.gov/pmc/articles/PMC6805518/

Author contributions
PB secured the funding. SC, AMV, AP, CP, RAA, and LM conducted the study and AMV, AP, SC, CP and RAA designed and performed the analysis. SC drafted the paper and PB, AMV, CP, RAA and LM critically revised it. The corresponding author attests that all listed authors meet the relevant criteria and no other meeting these have been omitted. SC acts as the guarantor for this study.

Data availability statement
Data are available upon reasonable request. The dataset used is available on reasonable request from the corresponding author.

Ethics approval statement
This study was approved by the North-West - Greater Manchester Central Research Ethics Committee (REC reference: 21/NW/0030), HRA and Health and Care Research Wales (HCRW) IRAS project ID: 281050. Participants gave informed consent to participate in the study before taking part.

Funding
National Institute for Health and Care Research Applied Research Collaboration Greater Manchester (NIHR ARC-GM). Grant number NIHR200174.

Disclaimer
The views expressed in this publication are those of the author(s) and not necessarily those of the National Institute for Health and Care Research or the Department of Health and Social Care.

Competing interests
None declared.

References

1. Galicia-Garcia, U., Benito-Vicente, A., Jebari, S., et al. Pathophysiology of Type 2 Diabetes Mellitus, *Int. Journal of Molecular Sciences*, 2020, Vol. 21:6275, doi:10.3390/ijms21176275
2. Chatterjee, S., Khunti, K. and Davies, M.J. Type 2 diabetes, *The Lancet*, 2017, Vol. 389, Issue 10085: P2239-2251, https://www.thelancet.com/journals/lancet/article/PIIS0140-6736(17)30058-2/fulltext#%20 (accessed 10/06/222)
3. Cities Changing Diabetes. Urban Diabetes: understanding the challenges and opportunities, 2016, available at: https://www.citieschangingdiabetes.com/content/dam/nnsites/ccd/en/knowledge-hub/reports/pdfs/CCD_BB_FULL_BOOK_FOR_CN_DTP.pdf (accessed 13/06/2022)
4. Mason, T., Whittaker, W., Dumville, J., et al. Estimating the 'Rule of Halves' for Greater Manchester: Initial analysis of data, NIHR ARC GM, University of Manchester, 2020: Manchester
5. Health Innovation Manchester. Cities Changing Diabetes: Mapping the challenge of type 2 diabetes in Greater Manchester, 2022, available at: https://healthinnovationmanchester.com/wp-content/uploads/2022/06/Cities-Changing-Diabetes-Manchester-report-final-June-2022.pdf (accessed 17/06/2022)

For peer review only - http://bmjopen.bmj.com/site/about/guidelines.xhtml

6. Volkmann, A.M., Hesseldal, L., Napier, D. et al. Urban Diabetes Priority Assessment, 2018, available at: https://www.citieschangingdiabetes.com/content/dam/nnsites/ccd/en/knowledge-hub/urban-diabetes-toolbox/pdfs/URBAN%20DIABETES%20PRIORITY%20ASSESSMENT_Intro%20Manual_Q22019_Screen.pdf (accessed 10/06/2022)
7. Browne, J. L., Nefs, G., Pouwer, F. et al. Depression, anxiety and self-care behaviours of young adults with Type 2 diabetes: results from the International Diabetes Management and Impact for Long-term Empowerment and Success (MILES) Study, *Diabetic Medicine*, 2014, Vol. 32:133–140, DOI: 10.1111/dme.12566
8. Kunasegaran, S., Beig, J., Khanolkar, M. et al. Adherence to medication, glycaemic control and hospital attendance in young adults with type 2 diabetes, *Internal Medicine Journal*, 2018, 48: 728–731 doi:10.1111/imj.13808
9. Nanayakkara, N., Pease, A.J., Ranasinha, S., et al. Younger people with Type 2 diabetes have poorer self-care practices compared with older people: results from the Australian National Diabetes Audit, *Diabetic Medicine*, 2018, Vol. 35 (8):1087-1095, DOI: 10.1111/dme.13660
10. Nanayakkara, N., Curtis, A.J., Heritier, S., et al. Impact of age at type 2 diabetes mellitus diagnosis on mortality and vascular complications: systematic review and meta-analyses, *Diabetologia*, 2020, 64 (2):275-287. doi: 10.1007/s00125-020-05319-w.
11. Tan, N.C., Barbier, S., Lim, W.Y. et al. 5-Year longitudinal study of determinants of glycemic control for multi-ethnic Asian patients with type 2 diabetes mellitus managed in primary care, *Diabetes research and clinical practice*, 2015, Vol. 110: 218-223
12. Cities Changing Diabetes. Urban Diabetes Priority Assessment, 2018, available at: https://www.citieschangingdiabetes.com/content/dam/nnsites/ccd/en/knowledge-hub/urban-diabetes-toolbox/pdfs/URBAN%20DIABETES%20PRIORITY%20ASSESSMENT_Intro%20Manual_Q22019_Screen.pdf (accessed 30/01/2022)
13. Napier, A.D., Nolan, J.J., Bagger, M., et al. Study protocol for the Cities Changing Diabetes programme: a global mixed-methods approach, *BMJ Open*, 2017, 7:e015240. doi: 10.1136/bmjopen-2016-015240
14. Cities Changing Diabetes. Bending The Curve On Urban Diabetes: New research approaches and innovative interventions for tackling diabetes in your city, 2017, available at: https://www.citieschangingdiabetes.com/content/dam/nnsites/ccd/en/knowledge-hub/reports/pdfs/14845_CCD17_BriefingBook_A4_09102017_LR.pdf (accessed 16/06/2022)
15. Cities Changing Diabetes. Urban Diabetes Toolbox, 2022, available at: https://www.citieschangingdiabetes.com/action-framework/urban-diabetes-toolbox.html (accessed 03/08/2022)
16. Pruneddu, A. and Zentner, M. QSortware, 2012, available at: www.qsortware.com.
17. Zabala, A. Qmethod: A package to explore human perspectives using Q methodology, *R Journal*, 2014, 6 (2), 163-173. https://doi.org/10.32614/rj-2014-032 available at: https://journal.r-project.org/archive/2014/RJ-2014-032/RJ-2014-032.pdf (accessed 05/08/2022)
18. Taylor, B., Henshall, C., Kenyon, S., et al. Can rapid approaches to qualitative analysis deliver timely, valid findings to clinical leaders? A mixed methods study comparing rapid and thematic analysis, *BMJ Open*, 2018, Vol. 8(10): e019993. doi:10.1136/bmjopen-2017-019993.

For peer review only - http://bmjopen.bmj.com/site/about/guidelines.xhtml

19. Vindrola-Padros, C., Chisnall, G., Cooper, S., et al. Carrying Out Rapid Qualitative Research During a Pandemic: Emerging Lessons From COVID-19, *Qualitative Health Research,* 2020, Vol. 30(14): 219-2204. doi.org/10.1177/1049732320951526.
20. Watts, S. and Stenner, P. Doing Q Methodological Research: Theory, Method and Interpretation, London, 2012: Sage Publications Ltd
21. ONS. Average household income, UK: financial year ending 2022, available at: https://www.ons.gov.uk/peoplepopulationandcommunity/personalandhouseholdfinances/incomeandwealth/bulletins/householddisposableincomeandinequality/financialyearending2022 (accessed 17/02/2023)
22. Walker, R.J., Smalls, B.L. and Egede, L.E. Social determinants of health in adults with type 2 diabetes—Contribution of mutable and immutable factors, *Diabetes research and clinical practice*, 2015, Vol. 110: 193-201

[Appendix 1 see separate file]

[Appendix 2 see separate file]

[Appendix 3 see separate file]

For peer review only

For peer review only - http://bmjopen.bmj.com/site/about/guidelines.xhtml

CCD/SC/Figure 1

"Understanding the health impact of social and cultural influences on people aged 18-40 living with type 2 diabetes: a mixed-methods study"

Figure 1. Example of a forced distribution grid (Watts & Stenner, 2012 p.17)

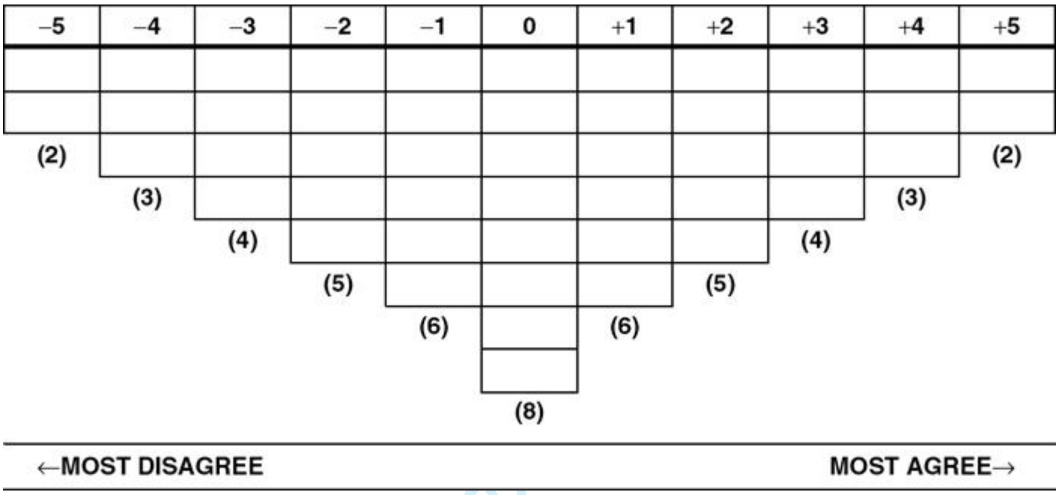

er review only

Appendix 1. Q-sort statements Greater Manchester factor array

	Factor 1	Factor 2	Factor 3	Factor 4	Factor 5
1 Diabetes is the least of my worries	-4	-4	0	-6	2
2 I worry about the cost of my diabetes	-3	3	-4	0	0
3 The way I make decisions about my healthcare is mostly dependent on how much money I have	-1	4	-2	3	-3
4 When it comes to food, I usually sacrifice quality for quantity	-1	-2	-4	5	6
5 Healthy foods are a luxury	-1	3	-1	1	1
6 Spending money on exercise classes or the gym is something I find difficult to do	1	5	-2	5	-1
7 Not having enough time makes people sick	1	2	-1	-4	-1
8 If I had more time, I'd exercise regularly	0	1	0	0	4
9 I just don't have the time to buy and cook healthy food	-4	1	-4	0	0
10 Diabetes support groups may be helpful, but I don't have time to participate	1	-1	2	6	-1
11 Taking care of others takes up most of my free time	0	5	-2	-2	2
12 I just don't have the skills to cook healthy meals	-3	-2	-5	1	3
13 For me, it's easy to learn about diabetes	4	0	3	-1	4
14 I think that diabetes or health-related support groups are pointless	-4	-3	0	-5	0
15 Where I live, fresh groceries are hard to get	-5	-3	-4	0	5
16 Living in the city is stressful and harmful to my health and wellbeing	-4	-1	1	-1	-5
17 I'd rather live elsewhere, but I have to live where I do because of work	-3	-4	4	2	-4
18 I am quite concerned about air pollution	0	0	0	3	-3
19 I think that where I live, it's not very safe to be out alone	-1	3	-5	-3	3
20 The climate where I live makes it really hard for me to want to walk or go somewhere by bicycle	-1	-1	-3	-1	-5
21 For me, it is important that I can get around by bike or on foot easily	0	0	-1	1	0
22 I like certain foods or dishes especially because they remind me of my childhood	1	1	1	4	-3
23 Cooking for those I love is a way of showing that I care for them	0	0	5	3	-1
24 When my family or my friends and I meet, we eat!	3	1	2	-1	-3
25 When I am by myself, I often eat food that is not very healthy	3	6	1	1	1
26 When I eat out together with friends, I usually end up eating unhealthy foods	6	4	3	-4	3
27 I often feel lonely	-5	2	2	6	2
28 To me, food is about more than just calorie intake	2	4	6	-1	3
29 Society puts a lot of pressure on women to be slim	5	5	-2	4	-3
30 I need my friends and social life to feel happy	4	0	-1	-2	-4
31 Being alone does not mean being lonely	4	2	3	0	-2
32 I tend to prioritise the needs of others over my own	3	6	0	1	-4
33 Men tend to be less interested in taking care of themselves than women	-2	-1	2	1	1
34 I have some health concerns that are more important to me than my diabetes	-2	0	1	-3	2
35 With today's medicine, diabetes is not a scary illness anymore	2	-2	3	-6	0
36 Diabetes is a disease of overweight people	-2	-5	2	-2	0
37 It is my fault that I now have diabetes	1	1	6	0	5
38 If I had known what having diabetes is like I would have tried harder to avoid becoming diabetic	2	3	0	3	-1
39 If I really had to, I could adjust to a healthy lifestyle	5	2	4	4	-2
40 I usually do what my doctor tells me to do	6	0	-1	-5	4
41 I avoid socialising because of my diabetes	-6	-3	-6	2	-1
42 I find it helpful to talk to other people about my diabetes	3	-2	-3	4	1
43 The modern world makes us ill	-1	1	3	1	-2
44 Feeling integrated with a community is important to me	1	-3	-1	3	1
45 Changes in my neighbourhood worry me	0	-1	0	0	4
46 I don't really trust health services or doctors	-6	-2	0	-5	0
47 Everybody just seems to be a bit bigger nowadays	1	0	5	0	2
48 My weight does not matter to me because I feel like a healthy person	-2	-6	-2	-3	5
49 Overweight people are often lazy	-2	-3	5	2	0
50 I am too embarrassed to go to the gym because of how I look	-1	2	0	-1	-5
51 Compared to others, I take good care of myself	0	-4	-3	-4	-2
52 I know who I can trust with my healthcare	2	0	-1	-2	1
53 I generally trust the government	0	-5	-6	-3	6
54 'Fitness freaks' have no joy in life	-3	-4	-3	2	2
55 I think that diabetes is inherited	2	1	1	-1	0
56 I think that diabetes is a death sentence	-5	-2	-2	2	-6
57 Most days, I feel good about my future	3	-1	-3	-2	3
58 At this point in my life, I feel like the choices I make are my own	5	4	4	-3	1
59 I get annoyed or upset with myself if I don't do enough for my health	2	3	1	5	-2
60 If I decide to be overweight or not exercise enough, that should be my choice	0	2	2	0	0
61 We owe it to society to be fit and healthy	0	0	1	-3	-4
62 Being unhealthy means you've given up on life	-3	-5	0	2	-1
63 Managing diabetes well is simply a matter of making the right choices	4	-1	4	-2	-2
64 My church is my rock	-2	-6	-5	0	-6
**NB: Distinguishing units are coloured**					

For peer review only

Appendix 2. Interviews and focus groups topic guide

Session	Areas to Be Covered	Example Questions/ Prompts (Not to be considered in any way exhaustive. Should be reformatted for ease of use in Focus Group or Interview session and language adapted if needed)
Introductory Session		
Open discussion around Q-Sort, content of statements, insights gained through procedure	Content of statements and nature of Q-sort	Principally unstructured discussion to give them an opportunity to voice what matters to them (and to allow participants to familiarise themselves with others in the Focus Groups).<br><br>If needed, specific sample statements may be presented as well as any qualitative comments already made. If needed, questions or prompts might include:<br>• How did you feel about X statement – is it easy to agree with/ disagree with? Why?<br>• Were there any statements that really spoke to you/ that really made you annoyed or sad? Why?<br>• Were there any statements that you did not understand or that you thought were just a bit silly? Why?<br>• Can you think of any statements that you thought were missing?<br>• The statements were based on interviews that we conducted with people in [CCD cities]. In what way do you think might their experience be similar or different to yours? Example?<br>• Were there any statements that made you reflect on how you yourself /your living with diabetes? Which ones?<br>• Did the sorting process make you think/ feel differently about yourself and your diabetes – if yes, why?<br>• Are there things we could have done differently with the Q-sort?
Discussion: Q-Sort findings		
Semi-structured discussion of findings with participants		This session provides an opportunity to deepen findings and to gather data for factor description and interpretation, as well as to identify any areas of relevance not covered by the statements. It should also be used to explore qualitatively areas of specific interest or priority. Finally, it is generally good practice to allow participants to comment on findings before finalising results.
	'Validity' of factors and exemplars	• How do you feel about [insert *defining* statements of groups]? Why?<br>• When you think about the groups (factors) that we have identified, do they seem typical/ plausible/ relevant/ well described? Why?<br>• How would you describe the various viewpoints associated with each group? Can you describe a person when you hear about these viewpoints? What would that person be like?

For peer review only

Appendix 2. Interviews and focus groups topic guide

		• What do you think of the names that we have given the groups?<br>• What do you think of the exemplars that we have described – do our descriptions make sense, do you think that this is informative/ accurate?<br>• Based on what we have presented to you, what would you say most characterizes/ differentiates the groups regarding their experience of diabetes? How does that relate to your own experiences?
	Relevance	• In what way do you think do the groups that we identified have different priorities/ abilities/ needs regarding health, wellbeing, and living with diabetes?<br>• In what way might our research findings (the groups and exemplars, description, interpretation) be useful when trying to improve diabetes care/ prevention?
	Barriers/ promoters	• How would you say do the different groups face different challenges regarding living with diabetes? What are those challenges; do you share them?<br>• What could be improved in terms of reducing [main stated barriers]?
	Social factors cultural determinants	Additional suggestion: provide list and description of social factors and cultural determinants and encourage discussion around which apply to what group; what their relevance and impact may be on each group, and how to ameliorate potentially negative impact.
Discussion: Diabetes in My City<br>**Semi-structured discussion around lived experiences of participants in Greater Manchester as relevant to health, wellbeing, and diabetes**		• Would you say that Greater Manchester is a good place to live for someone with diabetes? Why?<br>• Our physical activity level is important when it comes to living with diabetes. In what way does Greater Manchester make it easy or hard to be physically active? DO you have suggestions on how to change that?<br>• (If applicable: we have already discussed some things that may make living with diabetes in Greater Manchester difficult. Can you provide some more examples? How may we concretely address such issues?)<br>• If you could tell someone from [the city government/ local healthcare provider/ insurance (i.e. any potential stakeholders who may attend later session)] about something concrete they could do to improve your experience of living with diabetes, what would it be?

For peer review only

Appendix 3. Example RAP sheet

**Factor 1**	
**FGD/I participant(s):**<br>ID#<br>ID#	
**Q-sort comment(s):**<br>ID#<br>ID#<br>ID#<br>ID#	
**Analysis by:**	
**Diabetes journey**	
**Management**	
**GM lived experience**	
**Recommendations**	
**Notes (e.g. this interview a good source about x issue/standout quotations)**<br><br>ID#: “quote from transcript”<br><br>ID#: “quote from transcript”	

For peer review only

Consolidated criteria for reporting qualitative studies (COREQ): 32-item checklist

Please indicate in which section each item has been reported in your manuscript. If you do not feel an item applies to your manuscript, please enter N/A.

For further information about the COREQ guidelines, please see Tong *et al.*, 2017: https://doi.org/10.1093/intqhc/mzm042

No.	Item	Description	Section #
**Domain 1: Research team and reflexivity**			
Personal characteristics			
1.	Interviewer/facilitator	Which author/s conducted the interview or focus group?	p.6
2.	Credentials	What were the researcher's credentials? *E.g. PhD, MD*	p.1
3.	Occupation	What was their occupation at the time of the study?	p.1
4.	Gender	Was the researcher male or female?	N/A
5.	Experience and training	What experience or training did the researcher have?	p.6
Relationship with participants			
6.	Relationship established	Was a relationship established prior to study commencement?	p.5
7.	Participant knowledge of the interviewer	What did the participants know about the researcher? *E.g. Personal goals, reasons for doing the research*	N/A
8.	Interviewer characteristics	What characteristics were reported about the interviewer/facilitator? *E.g. Bias, assumptions, reasons and interests in the research topic*	N/A
**Domain 2: Study design**			
Theoretical framework			
9.	Methodological orientation and theory	What methodological orientation was stated to underpin the study? *E.g. grounded theory, discourse analysis, ethnography, phenomenology, content analysis*	p.5
Participant selection			
10.	Sampling	How were participants selected? *E.g. purposive, convenience, consecutive, snowball*	p.5
11.	Method of approach	How were participants approached? *E.g. face-to-face, telephone, mail, email*	p.5
12.	Sample size	How many participants were in the study?	p.7
13.	Non-participation	How many people refused to participate or dropped out? What were the reasons for this?	N/A
Setting			
14.	Setting of data collection	Where was the data collected? *E.g. home, clinic, workplace*	p.5-6
15.	Presence of non-participants	Was anyone else present besides the participants and researchers?	N/A

For peer review only - http://bmjopen.bmj.com/site/about/guidelines.xhtml

16.	Description of sample	What are the important characteristics of the sample? *E.g. demographic data, date*	p.5
Data collection			
17.	Interview guide	Were questions, prompts, guides provided by the authors? Was it pilot tested?	p.6
18.	Repeat interviews	Were repeat interviews carried out? If yes, how many?	N/A
19.	Audio/visual recording	Did the research use audio or visual recording to collect the data?	p.6
20.	Field notes	Were field notes made during and/or after the interview or focus group?	N/A
21.	Duration	What was the duration of the interviews or focus group?	p.6
22.	Data saturation	Was data saturation discussed?	N/A
23.	Transcripts returned	Were transcripts returned to participants for comment and/or correction?	N/A
**Domain 3: analysis and findings**			
Data analysis			
24.	Number of data coders	How many data coders coded the data?	p.13
25.	Description of the coding tree	Did authors provide a description of the coding tree?	N/A
26.	Derivation of themes	Were themes identified in advance or derived from the data?	p.7
27.	Software	What software, if applicable, was used to manage the data?	p.6
28.	Participant checking	Did participants provide feedback on the findings?	N/A
Reporting			
29.	Quotations presented	Were participant quotations presented to illustrate the themes / findings? Was each quotation identified? *E.g. Participant number*	p.7-9
30.	Data and findings consistent	Was there consistency between the data presented and the findings?	p.7-10
31.	Clarity of major themes	Were major themes clearly presented in the findings?	p.9-10
32.	Clarity of minor themes	Is there a description of diverse cases or discussion of minor themes?	p.11-12

When submitting your manuscript via the online submission form, please upload the completed checklist as a Figure/supplementary file.

If you would like this checklist to be included alongside your article, we ask that you upload the completed checklist to an online repository and include the guideline type, name of the repository, DOI and license in the *Data availability* section of your manuscript.

Developed from: Allison Tong, Peter Sainsbury, Jonathan Craig, Consolidated criteria for reporting qualitative research (COREQ): a 32-item checklist for interviews and focus groups, International Journal for Quality in Health Care, Volume 19, Issue 6, December 2007, Pages 349–357, https://doi.org/10.1093/intqhc/mzm042

For peer review only
